# A Systematic Review on Deep Structured Learning for COVID-19 Screening Using Chest CT from 2020 to 2022

**DOI:** 10.3390/healthcare11172388

**Published:** 2023-08-24

**Authors:** KC Santosh, Debasmita GhoshRoy, Suprim Nakarmi

**Affiliations:** 1 2AI: Applied Artificial Intelligence Research Lab, Vermillion, SD 57069, USA; 2 School of Automation, Banasthali Vidyapith, Tonk 304022, Rajasthan, India; debasmitaghoshroy@banasthali.in; 3 Department of Computer Science, University of South Dakota, Vermillion, SD 57069, USA; suprim.nakarmi@coyotes.usd.edu

**Keywords:** COVID-19, chest CT, deep structured learning, medical imaging

## Abstract

The emergence of the COVID-19 pandemic in Wuhan in 2019 led to the discovery of a novel coronavirus. The World Health Organization (WHO) designated it as a global pandemic on 11 March 2020 due to its rapid and widespread transmission. Its impact has had profound implications, particularly in the realm of public health. Extensive scientific endeavors have been directed towards devising effective treatment strategies and vaccines. Within the healthcare and medical imaging domain, the application of artificial intelligence (AI) has brought significant advantages. This study delves into peer-reviewed research articles spanning the years 2020 to 2022, focusing on AI-driven methodologies for the analysis and screening of COVID-19 through chest CT scan data. We assess the efficacy of deep learning algorithms in facilitating decision making processes. Our exploration encompasses various facets, including data collection, systematic contributions, emerging techniques, and encountered challenges. However, the comparison of outcomes between 2020 and 2022 proves intricate due to shifts in dataset magnitudes over time. The initiatives aimed at developing AI-powered tools for the detection, localization, and segmentation of COVID-19 cases are primarily centered on educational and training contexts. We deliberate on their merits and constraints, particularly in the context of necessitating cross-population train/test models. Our analysis encompassed a review of 231 research publications, bolstered by a meta-analysis employing search keywords (*COVID-19* OR *Coronavirus*) AND *chest CT* AND (*deep learning* OR *artificial intelligence* OR *medical imaging*) on both the PubMed Central Repository and Web of Science platforms.

## 1. Introduction

**Figure 1:**
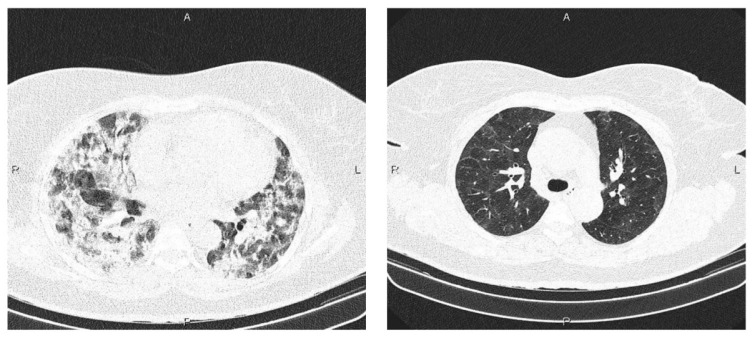
CT scan image data: Example image of COVID-19 positive CT scan (**left**) and non-COVID-19 CT scan (**right**) (courtesy: radiopaedia.org, rID: 85896).

As a result of severe acute respiratory syndrome coronavirus 2, the novel coronavirus (nCov), or simply COVID-19, emerged from Wuhan province, China (SARS-CoV-2) [1]. At the beginning of March 2020, the World Health Organization (WHO) classified it as a pandemic, and since then, the extent of the threat has been documented through confirmed cases and fatalities [2,3]. Following previous work [4], the spread of the COVID-19 virus can be expressed using the following transmission model, zi, b=Ai,b Ki,b Zi,b−2−Zi,b−8^ω, where *zi*, *b* represents the total number of infections in a country *i*, for a date *b*. *Ki*, *b* gives the population ratio still unaffected by COVID-19. *Ai*, *b* shows the transmitting rate and *Zi*, *b* is the cumulative number of subjects who have shown symptoms by the date *b*. The ω allows the rate of COVID-19 increase to be less than proportionate. This is only possible if ω < 1. The average serial interval is predicted to be around 4.5 days, given that interaction with infected people has remained constant throughout the interval [5,6]. Cough, headache, fever, muscle aches, shortness of breath, and dizziness are the most common symptoms of COVID-19 [7,8]. Symptoms may or may not be evident in some cases. The virus quickly weakens the subject’s immune system, resulting in death [9]. The WHO recommends the reverse-transcription polymerase chain reaction (RT-PCR) test, one of the numerous diagnostic procedures [10]. It is, however, time-consuming (typically taking 4–8 h) and costly. Artificial intelligence (AI)-guided tools can help speed up the screening process, particularly in areas with limited resources [11,12,13]. Since the beginning of 2020, several CADx systems have been proposed to use image data from chest X-rays (CXRs) and computed tomography (CT) scans to identify patients with COVID-19 infection. Apart from preventive approaches that use intelligent healthcare equipment [14,15], a few solutions have been presented to aid in diagnosing COVID-19 [16,17]. X-rays and chest CT scans are two common imaging modalities used for screening COVID-19 patients as they deliver consistent manifestations of COVID-19 [18,19,20] (see Figure 1).

Throughout the pandemic, numerous researchers have dedicated their endeavors towards the classification and detection of COVID-19 using CT scans and X-rays. While both modalities possess their respective advantages and disadvantages, there has been a prevailing inclination among authors toward CT scans. CT scans offer heightened sensitivity and visualization capabilities, albeit with the tradeoff of increased radiation exposure. The incorporation of deep learning (DL) algorithms has significantly streamlined clinical assessment and expert interpretation, rendering computer-aided diagnosis (CADx) models pivotal as supportive diagnostic tools in COVID-19 detection. Consequently, CADx imaging tools have garnered trust and now play a crucial role in COVID-19 screening. Within this study, our primary focus centered on CT scans, and the noteworthy contributions can be succinctly outlined as follows:

- Providing an account of accessible CT datasets and their utilization in deep learning (DL) for the classification of COVID-19;
- Conducting a performance evaluation that contrasts existing DL models through dataset utilization and methodological approaches;
- Implementing transfer learning (TF) and data augmentation (DA) techniques in the development of DL models;
- Proposing prospective directives for DL investigations within this particularly sensitive domain.

The remainder of the paper is organized as follows: Section 2 provides an overview of significance of the COVID-19 virus. The research scope and criteria for selecting articles are discussed in Section 3. Section 4 focuses on AI-guided medical imaging and presents a summary of COVID-19 screening investigations. This includes discussions on data collections and their sources (Section 4.1); the most used CT imaging tools (Section 4.2); the methodological contributions of DL-based methods/models in the years 2020, 2021, and 2022 (Section 4.3); performance comparisons (Section 4.4); dataset sizes (Section 4.5); transfer learning (Section 4.6); and data augmentation (Section 4.7). Finally, in Section 5, we conclude the paper and provide future guidelines. 

## 2. COVID-19: Background and Its Relevance

Schalk and Hawn [21] identified an ostensibly new respiratory illness in chicks in 1931 (between 2 days and 3 weeks old). We refer to a few papers, such as Frabricant (1998) [22] and Cook et al., for more specific progress on infectious bronchitis investigations (2021) [23]. SARS-CoV [24] was discovered in China between 2002 and 2003. Approximately 8000 people were infected during these years, with a 9.5% fatality rate. Bats or civet cats were suspected of being the cause of the disease [25]. MERS-CoV—a version of the coronavirus—was discovered in 2012 in Saudi Arabia [26]. In 2019, we had approximately 2500 MERS-CoV infections, with a 30% death rate [27]. The transmission agents were thought to be camels this time (dromedaries) [28]. SARS-CoV-2 is one of the oldest known viruses, infecting humans through a common cold. The virus is transmitted via inhalation or ingestion of droplets produced by coughing and sneezing. The viral structure comprises roughly 30,000 nucleotides and contains four structural proteins: spike, membrane, envelope, nucleocapsid [29,30,31], and various nonstructural proteins. N-protein (viral positive strand RNA) is also found in the protein shell or capsid. This strand acts as a parasite in human cells that proceeds to replication and transcription. Lung screening for nCoV looks like influenza-associated pneumonia in terms of analysis [32,33].

## 3. Study Scope and Selection Criteria

**Figure 2:**
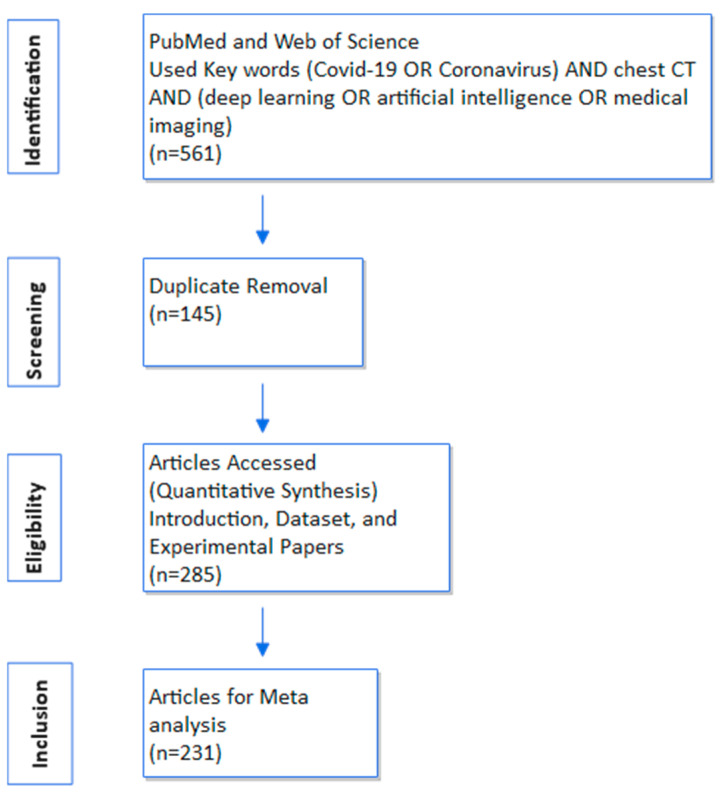
Workflow represents different systematic review phases (source: PRISMA criteria [34]).

Before commencing our review, let us adhere to a systematic workflow delineating multiple stages, including identification, screening, eligibility, and inclusion criteria, as illustrated in Figure 2. To identify relevant studies, we employed search keywords (*COVID-19* OR *Coronavirus*) AND *chest CT* AND (*deep learning* OR *artificial intelligence* OR *medical imaging*) on both the PubMed Central Repository and Web of Science platforms. Following this, duplicate entries were eliminated. Specifically, we focused on experiment-based research publications utilizing deep learning (DL) models/algorithms, limited to the year 2020 for publication. To ensure rigor, we excluded preprint articles from arXiv, medRxiv, and TechRxiv due to their non-peer-reviewed status. Our assessment encompassed diverse aspects, such as dataset characteristics (size and source), technical intricacies (DL models), and corresponding performance metrics, enhancing the potential for comprehensive meta-analysis. Our primary objective is not solely to delineate performance rankings among research articles, but rather to gauge the progress achieved since the onset of the pandemic. Furthermore, our evaluation extends to critical considerations like dataset scale, data augmentation techniques, and the applicability of transfer learning methodologies.

## 4. AI for Medical Imaging for COVID-19

By extracting distinctive features, AI-guided technologies can streamline complex data representations, making decision making achievable. They lead to a wide range of applications, including drug discovery [35], innovative healthcare [36], biomedicine [37], and medical image analysis [38]. Deep learning algorithms are prevalent in all cases [39,40]. However, we still have challenges in developing a clinical screening tool that considers various variables. Recent studies have discussed the value of artificial intelligence (AI) in the prognostication and diagnosis of medical images [41,42]. According to the WHO, COVID-19 is a global public health emergency and the most significant test we currently face [43,44,45]. Research on supervised algorithms for COVID-19 identification (classification) and segmentation has been the primary focus since the first quarter of 2020 [46,47,48]. Few promising investigations concentrate on dual-sampling attention networks [49]. Interestingly, approaches that fall under the purview of unsupervised learning outperform supervised ones, even though most rely on supervised techniques [50,51,52]. In what follows, we first provide CT scan datasets and their respective sources. The research articles that used CT scans are then reviewed for their methodological contributions. Although we acknowledge thousands of research articles published in the year 2020 and 2021, our study is limited only to experiment-based (with DL models), peer-reviewed articles other than preprints: medRxiv, TechRiv, and arxiv by using exact search keywords in PubMed Central Repository and Web of Science (Section 3).

### 4.1. Dataset and Availability

Plenty of CT scan-based datasets are available in the literature to identify COVID-19. Almost all the previously stated peer-reviewed studies employed various datasets in their setup and system designs. Before training/validating their systems, the general concept is to acquire datasets (private or public access) from internet sources or prepare with their method. Therefore, understanding and determining the best approaches from the available reports is physically challenging. Data unavailability is a widespread issue for computational scientists as many datasets are required for their machine/deep learning model. Unlike other established infectious diseases, the absence of clinically annotated data is still present for COVID-19. However, as the number of COVID-19 cases grows, the datasets are regularly updated (see Table 1).

C1. The COVID-CT dataset consists of 746 CT scans, 349 of which are COVID-19-positive cases.
C2. The COVID-19 dataset contains 829 CT scans, of which 373 are COVID-19-positive cases.
C3. Large COVID-19 CT scan dataset comprises 2282 COVID-19-positive CT scans and 12,058 CT scans.
C4. SARS-CoV-2 dataset has 2482 CT scans with 1252 COVID-19-positive cases.
C5. COVID-19 open research dataset (CORD-19) consists of 3439 CT scan images, 98 of which are COVID-19-positive cases.
C6. SIRM COVID-19 database contains 100 CT scans.
C7. COVID-19 BSTI imaging dataset details are not available.
C8. Radiopaedia dataset consists of 36,559 CT scans, where 3520 are COVID-19-positive cases. It is the largest dataset.
C9. MosMeddata dataset is composed of 1110 CT scans.
C10. COVID-19 dataset contains 521 CT scans, where 48 are COVID-19-positive cases.
C11. COVID-CS dataset contains 3855 CXRs, where 200 are COVID-19-positive cases.
C12. Medical imaging databank in the Valencia region medical image bank (BIMCV) COVID-19 dataset consists of 1311 COVID-19-positive CT scans and 6687 CT scans.
C13. COVID-19-CT-CXR dataset contains 1327 CT scans, and the author has not disclosed the number of positive cases.
C14. Larxel dataset is composed of 20 COVID-19-positive CT scans.
C15. Large COVID-19 CT scan slice dataset consists of 7593 COVID-19 positive CT scans and a total of 17,102.
C16. Extensive COVID-19 X-Ray and CT Chest images dataset has 17,099 CT scans, including 5427 COVID-19-positive cases.
C17. CF dataset contributes 19,685 images with 4001 COVID-19-positive cases.
C18. COVID-19 image dataset contains 22,873 CT scans, where 3520 are COVID-19-positive.
C19. China consortium of chest CT image investigation (CC-CCII) Dataset comprises 4178 CT scans, 1544 of which are COVID-19-positive.
C20. COVID-CT-MD dataset is private.
C21. Deep Covid dataset consists of 5000 CT scans. They have not disclosed the number of positive cases.
C22. CT scan for COVID-19 dataset contains 13,980 CT scans, where 4001 are COVID-19-positive cases.
C23. Covid Chest Xray and CT images dataset consist of 144 CT scans, where 118 are COVID-19-positive cases.
C24. Harvard dataverse dataset contains 4172 CT scans with 2167 COVID-19-positive cases.
C25. COVIDx CT is the largest dataset with 431,205 CT scans, including 316,774 COVID-19-positive cases.

### 4.2. CT Imaging Tools

DenseNet is a new convolution network architecture proposed by G. Huang, Z. Liu, and K. Weinberger in the paper “Densely Connected Convolutional Networks” [53]. They reported that the proposed architecture achieved high performance with four benchmark tasks: CIFAR-10, CIFAR-100, SVHN, and ImageNet. 

The VGG network is a convolution neural network model implemented by K. Simonyan and A. Zisserman in the paper “very deep convolutional networks for large-scale image recognition” [54]. This architecture was beneficial for classification accuracy, and the highest performance accuracy was archived with 14 million images belonging to 1000 classes. 

InceptionNet is a deep convolution neural network architecture proposed by C. Szegedy and others in the paper “Going deeper with convolutions” [55]. This proposed approach was experimentally verified on the ILSVRC 2014 classification and detection challenges, significantly outperforming the current state of the art. 

A.G. Howard proposed MobileNets architecture in the paper “MobileNets: Efficient Convolutional Neural Networks for Mobile Vision Applications” [56]. This architecture based on depth-wise separable convolutions, and it provided an effective result for a wide variety of tasks. 

ResNet is a residual learning framework proposed by K. He and others in the paper “Deep Residual Learning for Image Recognition” [57]. This architecture is easy to train as compared to the deep neural network. The result came from this architecture winning 1st place on the ILSVRC 2015 classification task. 

Y. Le Cel proposed a CNN architecture in the paper “Backpropagation Applied to Handwritten Zip Code Recognition” [58]. This architecture is flexible to network design and is used in image classification. 

UNet: O. Ronneberger introduced UNet architecture in the paper “U-Net: Convolution Networks for Biomedical Image Segmentation” [59]. This architecture achieved excellent performance on different biomedical segmentation applications.

### 4.3. Identification of COVID-19 Using CT Imaging Tools (2020–2022)

In this particular context, our focus has been on investigating the practical application of CT imaging as a valuable diagnostic tool for accurately detecting instances of COVID-19. This investigation has been concentrated on the timeframe spanning from 2020 to 2022 (See Table 2).

#### 4.3.1. 2020

Ni et al. [60] used the DL approach to diagnose COVID-19, and 96 positive cases were considered in training. Their AI-based system performance was compared with radiologist residents based on per-lobe lung- and per-patient-level. They reported that the sensitivity was superior to the AI model for per-lobe lung and per-patient levels, respectively. Hu et al. [61] introduced a self-trans network to identify COVID-19 where DenseNet169 architecture was used. They compared the model performance with ResNet50 and the self-trans approach, and 86% accuracy was reported. Loey et al. [62] used five DL models, namely AlexNet, VGGNet16, VGGNet19, GoogleNet, and ResNet50, to differentiate between COVID-19 and non-COVID-19 patients. They reported that ResNet50 outperforms others with an accuracy of 82.91%. Song et al. [63] developed a BigBiGAN model to identify COVID-19 pneumonia from other pneumonia with 93 positive cases. The model performance was estimated using the AUC value with internal tests and external validation. They reported an AUC value of 85% for external validation and 97% for internal testing. Chaganti et al. [64] conducted a retrospective study to determine CT abnormalities and severity scores based on the DL model. A total of 901 CT scans with 431 positive cases were used. Three measurement indices, such as Pearson and Kendall’s correlation and chi-square, helped identify the severity. 

Singh et al. [65] proposed a mode-based CNN and competitive model to classify COVID-19-infected patients as positive vs. negative. The model performance was compared with three N, CNN: ANN. They reported that the proposed model outperforms with a good accuracy rate. Ning et al. [66] used the DL algorithm to discriminate between negative mild and severe cases of COVID-19. They reported the AUCs of 0.944, 0.860, and 0.884 for each class. Jaiswal et al. [67] implemented the DenseNet201 model and compared the performance with three different DL models. They reported that the maximum accuracy of 99.82% was achieved using DenseNet201. Babukarthik et al. [68] implemented a model based on the GDCNN algorithm to classify normal vs. COVID-19. The proposed model’s performance was compared with five different DL models. They reported that the proposed model outperforms other models with an accuracy of 98.84%. Mohammed et al. [69] developed the RestNet+ model to classify COVID-19 vs. other pneumonia. They reported proposed model accuracy of 77.6%. Han et al. [70] proposed attention-based deep 3D multi-instance learning (AD3D-MIL), compared it with traditional multi-instance learning, and reported the proposed model accuracy of 97.9%. Jiang et al. [71] used AI models for the diagnosis of COVID-19 by use of a cGAN structure image that can generate realistic city images with two types of infections: ground-glass opacity and consolidation. They reported that the model achieved an accuracy of 98.37% for COVID19+ vs. COVID-19−. Gunraj et al. [72] introduced the COVIDx-CT model to identify COVID-19 vs. normal vs. pneumonia. The proposed model performance was compared with the ResNet50, NASNet-A-Mobile, and EfficientNetB0 models. They reported that the model achieved an accuracy of 99.1%. 

Fan et al. [47] developed the Inf-Net (covid lung CT infection segmentation) and semi-Inf-Net model (cutting-edge segmentation). These models could detect objects with low-intensity contrast between infected and normal tissues. They reported specificity and sensitivity of 97.55% and 86.75%, respectively. Mishra et al. [73] applied a deep CNN-based approach including five models: VGG16, InceptionV3, ResNet50, DenseNet121, and DenseNet201 decision and developed a new model. They reported a model AUC value of 88.3%. Javor et al. [74] devised a DL model based on the ResNet50 model architecture to classify covid patients with 6868 CT images. They reported an AUC value of 95.6%. Silva et al. [75] devised a model, Efficient Covid Net, along with a voting-based approach and cross-dataset analysis. The proposed model achieved an accuracy of 87.68%. Pathak et al. [76] used ResNet50 architecture to identify COVID-19 and reported an accuracy of 93.01%. Wu et al. [77] conducted a multicenter study with 294 COVID-19-positive cases. They designed a model using a DL network trained using multi-view images, and 76% accuracy was reported. Peng et al. [78], DenseNet121 was pretrained on ImageNet to create a classification model. They reported that the model achieved the highest performance of 89.1 in AUC. Qian et al. [79] used 2D-CNN architecture to design a DL model to classify COVID-19 patients. They reported that the model performed well. Li et al. [80] used the DL model to detect COVID-19 accurately and reported that the model achieved an AUC of 96%. They claimed this proposed model could also detect community-acquired pneumonia and other lung diseases from COVID-19 cases. Lessmann et al. [81] used AI techniques to identify COVID-19, and diagnosis performance was compared with the radiologic observer. The proposed model obtained an AUC score of 95% and claimed that their proposed model could easily assess the severity of the disease. 

Jin et al. [82] proposed an AI system for rapidly identifying COVID-19 from influenza A/B, nonviral community-acquired pneumonia (CAP), and non-pneumonia subjects. They used 2D deep CNN, whose backbone was ResNet152, and the reported accuracy was 94.98%. Jamshidi et al. [83] introduced a deep CNN network to identify COVID-19 patients. They reported that the proposed network achieved an accuracy of 98.49%. Wang et al. [84] devised the 3D-DeCoVNet model to identify COVID-19, and the proposed model reported 90.01% accuracy. Zhang et al. [85] proposed the CoVNet model based on 3D CNN, and an AUC value of 95.9% was reported. Lai et al. [86] used DCNN architecture to identify COVID-19-NCIP. They reported that the proposed model achieved an AUC value of 91%. Liu et al. [87] used four DL models: DenseNet121, DenseNet169, DenseNet201, and the baseline model VGG19. They compared the model performance in weighted vs. unweighted form. The average weighted and unweighted AUC values for the DL models were 76.09% and 72.77%, respectively. Panwar et al. [88] used CNN architecture to detect COVID-19 vs. normal vs. pneumonia. They reported an accuracy of 95.61% for classification between COVID-19 vs. normal. Misztal et al. [89] used five pretrained DL models, ResNet18, ResNet50, DenseNet169, wideResNet50, and DenseNet121, to classify NC vs. COVID-19 vs. bacterial pneumonia vs. viral pneumonia. The authors created a new dataset named radiograph image data stock to increase the efficiency of COVID-19 identification. They concluded that the newly designed dataset performed better with binary and multiclass classifiers. Amyar et al. [90] devised a multitask DL model and compared it with CNNs like UNet. They reported that the model obtained an accuracy of 94.67%, and 88% accuracy was reported for the segmentation task. Polsinelli et al. [91] proposed a CNN design based on the model of the SqueezeNet to classify COVID-19. They compared the model performance with the original SqueezeNet and 85. 03% accuracy was obtained via the proposed model. 

In [92], Ko et al. implemented the FCONet model based on ResNet50 architecture to diagnose COVID-19 pneumonia. They compared the model performance with Xception, InceptionV3, and VGG16. Of all, the ResNet50-based model achieved maximum accuracy of 96.97%. El-Bana et al. [93] introduced a new DL model named TL InceptionV3 to identify COVID-19 and compared it with seven states of art approaches for two classes. They reported that TL InceptionV3 attained maximum performance with an accuracy of 99.5%. Wang et al. [94] proposed 3D-Unet model to classify COVID-19 vs. viral pneumonia vs. normal. They reported that the model obtained an accuracy of 93.3% for the classification tasks. Deng et al. [95] formulated the Keras-related DL approach for COVID-19 detection. They used SVM and CNN algorithms to compare the classification performance. Finally, 75% accuracy was obtained by the proposed model (PTVGG16). Hu et al. [96] designed NTS-NET model to identify COVID-19 vs. NP vs. CAP. They reported that the proposed model achieved an accuracy of 84.3% for COVID-19 Identification. Li et al. [80] developed a 3D deep learning framework called COVNET. The proposed model provided an AUC value of 96% for detecting COVID-19 vs. CAP vs. NP. Xu et al. [97] used ResNet architecture to differentiate COVID-19 vs. IAVP vs. healthy cases. They reported that 87% accuracy was obtained for classification. Wang et al. [98] introduced a model named as Covid19Net based on DenseNet architecture and reported an 85% AUC score. Kang et al. [99] applied multi-view representation learning to identify the relationship between COVID-19 vs. CAP. They reported an accuracy of 95.5% achieved by the proposed approach. Chen et al. [100] deployed UNet++ architecture and designed a model to identify COVID-19. They reported that the proposed model achieved an accuracy of 98.85%. Bai et al. [101] devised a system based on DNN Efficient NetB4 architecture, and 96% accuracy was reported. Zhu et al. [102] proposed a model to assess the disease severity using VGG16 network architecture. Benbrahim et al. [103] used InceptionV3 and ResNet50 architecture to identify normal vs. COVID-19 patients. The model has attained an accuracy of 99.01%. Sharma et al. [104] proposed a model based on ResNet architecture and Grad-cam, which achieved an accuracy of 87.6%. 

In 2020, we found 47 articles for identifying COVID-19 using different types of datasets, where 23 worked on the private dataset. Most authors used CNN, ResNet, DenseNet, Aleand xNet, and DCNN architectures and compared results with available DL methodology. A few of them modified the existing structure of DL architecture and proposed a new model with different names like Covid19Net, CoVNet, DecoVNet, and so on. In addition to these, we found a few articles where authors also concentrated on classifying and segmenting COVID-19 patients from influenza, nonviral community-acquired pneumonia, and nonpneumonia diseases.

#### 4.3.2. 2021

Ibrahim et al. [105] used four deep learning architectures, namely VGG19-CNN, ResNet152V2, ResNet152V2 + GRU, and ResNet + Bi-GRU, to classify COVID-19 vs. normal. The maximum accuracy of 98.05% was achieved by the VGG19-CNN model. The authors claimed this model could also identify lung cancer and pneumonia, the first deep learning model in the literature. Goncharov et al. [106] implemented a multitask spatial-1 model to identify COVID-19 vs. normal class using severity score. The model outperforms other approaches and achieved an AUC score of 0.97 ± 0.01 between COVID-19 and healthy control. Additionally, the Spearman correlation method was used to find severity quantification. Zhang et al. [107] devised a new five-layer DCNN model with 3CB + 2FCBs for COVID-19 diagnosis. The implemented method was compared with six deep learning algorithms: RBFNN, K-ELM, ELM-BA, 6L-CNN-F, GoogleNet, and ResNet18. A model accuracy of 93.64% was obtained with stochastic pooling, providing better performance than average and max pooling. Song et al. [108] implemented the DRE-Net model to identify COVID-19 vs. healthy people among 274 patients. They compared their model performance with three DL models, namely VGG16, DenseNet, and ResNet. The maximum accuracy of 86% was achieved by DRE-Net. Additionally, this model could also identify bacterial pneumonia patients due to covid with 93% accuracy. Yao et al. [109] conducted a retrospective multicenter study to identify mild COVID-19 pneumonia by implementing CNN-based DL model. They also compared the model performance with the radiologist. The overall sensitivity and specificity were 91.5% and 90.5%. Acar et al. [110] used nine DL models, namely VGG16, VGG19, Xception, ResNet50, ResNet50V2, InceptionV3, InceptionResNetV2, DenseNet121, and DenseNet169. They used internal and external datasets to access each model’s performance with normal and augmented datasets. Finally, accuracy was improved from 3% to 9% for each DL model. Ravi et al. [111] used a stacked ensemble meta-classifier and deep learning-based feature fusion approach in CXR and CT images to classify COVID-19 vs. non-COVID-19 samples. They performed a comparison study with existing available pertained CNN models. Finally, a maximum accuracy of 99% was reported using CT data. 

Chen et al. [112] used different ResNet architectures to classify normal vs. COVID-19 vs. other pneumonia. Of all, ResNet50 provided the best classification accuracy of 91.21%. They also compared their result with the radiologist, and the proposed model achieved an overall accuracy of 89.01%. Huang et al. [113] implemented a FaNet network to classify normal vs. COVID-19 with 416 samples. They compared the result with six different models: AlexNet, ResNet, MobileNet, VGG, SENet, and DenseNet. Finally, 98.28% accuracy was reported for diagnosis assessment via FANet. The authors also claimed that their proposed model could assess the severity of COVID-19 with an accuracy of 94.83%. Jangam et al. [114] utilized stack ensemble techniques to develop an automatic COVID-19 detection system and compared the performance with four pretrained DL models. They reported 84.73%, 99%, and 90.75% accuracy for three different datasets. Singh et al. [115] implemented a MobileNet model that takes lesser time for covid classification. They compared their model performance with three DL model architectures: the proposed model reported an accuracy of 96.40%. Alirr et al. [116] devised FCN using Unet architecture for COVID-19 infection vs. lung segmentation that was evaluated qualitatively and quantitatively with a diverse dataset. They reported that the proposed model has a sensitivity and specificity of 82.2% and 95.1%, respectively. Kundu et al. [117] established a fully automated DL model for differentiating COVID-19 vs. non-COVID-19 patients. The proposed model performance was compared with three DL models: InceptionV3, ResNet34, and DenseNet201. They reported a proposed model accuracy of 97.81%. Saad et al. [118] implemented DFC model to identify COVID-19 vs. non-COVID-19 samples. The model performance was compared by 14 other different methods. They reported a model accuracy of 98.9%. Fung et al. [119] implemented an SSInfNet model that utilized DL to support rapid COVID-19 diagnosis and reported an AUC of 98.66%. Tan et al. [120] implemented the VGG16 model to classify COVID-19 with an accuracy of 98%. Lascu et al. [121] utilized ResNet101 architecture to classify COVID-19 among four class labels. They reported an accuracy of 94.9%. Lassau et al. [122] built an AI model to determine the severity score to diagnose severe evolution for COVID-19. They compared their proposed approach to an existing severity score of 11, and performance improvement was reported. Pan et al. [123] determined the correlation between the conventional CT scoring system and the proposed DL-based quantification. They reported that the proposed DL quantification correlated with conventional CT scoring and demonstrated a potential benefit in estimating COVID-19 severity. Yan et al. [124] developed a Fast.AI ResNet framework to differentiate COVID-19 vs. pneumonia vs. normal. The authors compared the model performance with three DL models: VGG16, DenseNet121, and ResNet152. Finally, the maximum accuracy was achieved by ResNet50 with Fast. AI. Shalbaf et al. [125] used 15 pretrained CNN architectures and developed an ensemble model using majority voting criteria. Rahimzadeh et al. [126] proposed a new feature pyramid network with the ResNet50V2 model to classify COVID-19. The model performance was compared with two DL models: Xception and Resnet50V2. The proposed model’s accuracy of 98.49% was reported. Lee et al. [127] devised the DeteCT model to automatically predict COVID-19+ from COVID-19-, pneumonia, and normal controls. They reported that the proposed model AUC value is more than 80% on most test sides. Mishra et al. [128] used a deep learning algorithm to diagnose COVID-19. The authors also worked on finding ANN’s severity index of covid infection. They reported an accuracy of 99%. Zhang et al. [129] proposed an improved segmentation model called the residual attention U-shaped Network. The model was evaluated using 100 scan datasets resulting in mIoU and dice coefficient values of 84.5% and 73.4%, respectively. 

Barbosa et al. [130] conducted a retrospective study to differentiate between COVID-19 vs. non-COVID-19 patients. They concluded that the CNN-trained model achieved an expert level of accuracy in quantifying COVID-19 airspace disease. Zhao et al. [131] developed a new approach, an image deformation-based segmentation model, SP-V-Netbased. They reported that the model achieved an accuracy of 94.60% for COVID-19 classification. Jadhav et al. [132] proposed a COVID-19-view by incorporating a novel DL method to classify the patients into positive and negative COVID-19 cases. This model can also be used for lung segmentation, lesion localization, and detection. They reported an accuracy of 95.2%. Guiot et al. [133] developed a detection model on 181 COVID-19+ cases using VGG16 architecture. They reported the proposed model accuracy of 85.18%. Yao et al. [134] devised a model named as CSGBBNet for the classification of COVID-19 and reported an accuracy of 98.49%. Singh et al. [135] designed a DL-based model for detecting COVID-19. The model performance was compared with four DL models: Gen-ProtoPNet, NP-ProtoPNet, ProtoPNet, and VGG16. They reported that the proposed model outperformed with an accuracy of 99.29%. Zhu et al. [136] used ResNet50 to classify normal vs. COVID-19 by 1357 confirmed positive cases. The model performance was compared with VGG19 +GoogleNet architecture-based DL model. They reported that the model achieved an accuracy of 93%, which is better than other DL models. Kuchana et al. [137] developed a model based on UNet architecture for two segmentation tasks: lung spaces and COVID-19 anomalies. The model performance was compared with standard UNet and attention UNet. They reported that the proposed model obtained a F1 score of 97.31%. Khalifa et al. [138] used DL semantic segmentation architecture for COVID-19 lesion detection. The model consists of an encoder and decoder component. They reported that the model achieved 99.3% accuracy. Bhuyan et al. [139] developed a model to detect COVID-19, classification, and segmentation. The model performance was compared with and without mass segmentation via different *k*th validation techniques. They reported that the proposed model (FrCN) accuracy was optimal with mass segmentation and fourth-fold validation techniques. Heidarian et al. [140] proposed a COVID-Fast model based on CNN to detect COVID-19 and non-COVID-19 cases. They reported the proposed model accuracy of 90.82% for COVID-19 identification. Ahsan et al. [141] implemented six deep CNN models: VGG16, MobileNetV2, InceptionResNetV2, ResNet50, ResNet101, and VGG19 with 400 CT images. They reported that MobileNetV2 outperforms with an accuracy of 98.5%. Zhang et al. [142] implemented a GARCD model to classify COVID-19+ and normal. The performance was compared with four models: ResNet, GADCD, VGG19, and DenseNet. They reported GARCD model achieved an optimal AUC value of 98.7%. Chaddad et al. [143] used deep CNN architecture (AlexNet, DenseNet, GoogleNet, NASNet-Mobile, ResNet18, and DarkNet) to classify COVID-19 vs. normal. The proposed model has achieved an accuracy of 82%. They also claimed that the proposed model could classify COVID-19+ or COVID-19- from X-ray images. Yousefzadeh et al. [144] implemented a deep learning-based covid classification model named ai-corona. They reported proposed model performed well, and the average AUC was 98%. Chen et al. [145] proposed a model based on few-shot learning in ResNet50 architecture to classify COVID-19 vs. non-COVID-19 with few samples. The performance of the new algorithm-based model was compared with three different methods: ResNet152, DenseNet161, and VGG16. They reported accuracy and AUC value of 86.8% and 93.1%, respectively. Munusamy et al. [146] developed a FractalCovNet model consist of UNet architecture to classify COVID-19. They compared the model performance with ResNet50, Xception, InceptionResNetV2, VGG16, and DenseNet. They reported the proposed model accuracy of 99%. 

Wang et al. [147] developed a CCSHNet model based on a DCFDCA algorithm to classify COVID-19. The proposed model performance was compared with 12 existing models. They reported that the model outperformed. Jiang et al. [148] used five CNN models, namely DenseNet169, InceptionResNetV2, InceptionV3, ResNet50, and VGG16, to identify the effectiveness of the dataset. They reported that a maximum accuracy of 96% was obtained using synthetic data. Hu et al. [149] proposed DSN-SAAL model, and performance was compared with seven models: VGG16, ResNet50, DenseNet169, Self-Trans, contrastive COVIDNet, transfer CheXNet, and cross-dataset analysis. They reported that the proposed model outperforms all used datasets. The achieved average accuracy of the proposed model is 95.43%. Jingxin et al. [150] used the DL approach based on ResNet50 and compared it with Mark R-CNN, UNet. They reported an accuracy of 97.83% via Ours-SP. Balaha et al. [151] developed a covid detection model named CovH2SD based on VGG16 architecture. A total of nine experiments were performed (ResNet50, ResNet101, VGG16, VGG19, Xception, MobileNetV1, MobileNetV2, DenseNet121, and DenseNet169) on CT images. Of all, the best result was achieved by VGG16. Turkoglu et al. [152] proposed a model named as MKs-ELM-DNN based on DenseNet201 architecture. They compared the performance of six models (AlexNet, GoogleNet, VGG16, MobileNetV2, ResNet18, and InceptionV3). The maximum accuracy of 98.36% was achieved by DenseNet201. Ahamed et al. [153] proposed a model based on a modified ResNet50V2 architecture to differentiate between COVID-19, normal controls, and viral and bacterial pneumonia. The model performance was compared with nine pre-trained CNN models and reported an accuracy of 99.99% for two-class cases (COVID-19/normal). Pathan et al. [154] devised a COVID-19 classification model that deployed an ensemble of five CNNs architecture for feature extraction, and extracted features were again selected by a binary grey wolf optimizer. Model performance was compared with four existing studies, and 96% accuracy was reported. Cruz et al. [155] implemented a model based on an ensemble method using six pretrained DL models: VGG16, ResNet50, wideResNet50-2, DenseNet161, DenseNet169, and InceptionV3. They compared the model performance with eight different models. The maximum accuracy of 86.70% was achieved by the proposed ensemble method. Hasan et al. [156] designed a model based on two fundamental deep learning models, VGG16 and VGG19, for the classification of COVID-19. The model performance was compared between original vs. modified images. They reported that 87.37% accuracy was achieved using original images, whereas 90.14% accuracy was reported for modified images. Basset et al. [157] devised a model to classify COVID-19 based on lung area infection segmentation. They compared their model performance against other studies: R2UNet, CE-Net, and CPFNet. The proposed model outperforms with 96.80% accuracy. 

Fu et al. [158] designed and compared a classification model named DenseAnet with seven models. They reported that the maximum accuracy of 90.27% was achieved using DenseAnet. Aslan et al. [159] proposed a hybrid model based on mAlexNet+ BiLSTM architecture, and 98.70% accuracy was reported. Kundu et al. [160] used the Sugeno fuzzy integral ensemble of four pretrained deep learning models, namely VGG11, GoogleNet, SqueezeNet v1.1, and wideResNet50-2. The proposed model achieved an accuracy of 98.93%. Müller et al. [161] used 3D UNet architecture to classify COVID-19+ and normal slices, and the performance was estimated using the DSC score. They reported that the proposed model performed well compared with existing studies. Li et al. [162] developed a deep learning model called CheXNet and evaluated their proposed method with other existing methods. Finally, maximum accuracy was achieved by the proposed DL model with an accuracy of 87%. Zhang et al. [163] created an end-to-end multiple-input deep convolutional attention network based on a convolution attention module. The model provided better outcomes than eight state-of-the-art approaches. They reported that the model obtained an accuracy of 98.02%. Xu et al. [164] proposed two models: CARes-UNet and semi-CARes-UNet. They compared the model’s performance with nine existing models, and the semi-CARes-UNet model provided the best outcome close to ground truth. Mondal et al. [165] proposed a DL model, namely CO-IRv2, to classify COVID-19. They used three optimizers and achieved 96.18% accuracy for binary classification. Chen et al. [166] developed an ensemble CNN (covid-CNN) model based on five pretrained DL architectures: VGG19, ResNet101, DenseNet201, InceptionV3, and InceptionResNetV2. They compared the proposed model’s performance with the existing CNN model. Covid-CNN obtained the maximum accuracy of 96.7%. Alshazly et al. [167] considered seven CNN networks: SqueezeNet, Inception, ResNet, ResNeXt, Xception, ShuffleNet, and DenseNet, and their performance was compared. ResNet101 and DenseNet201 performed best, with an accuracy of 99.4% and 92.9%, respectively. 

Voulodimos et al. [168] proposed a few-shot UNet model and compared it with the conventional UNet model. They observed that the proposed model F1 score was improved by 5.394 ± 3.015% and the increment of precision and recall value by 1.162 ±2.137% and 4.409 ± 4.790%, respectively. Khan et al. [169] proposed a model MC-SVM along with optimal deep model features. They reported that the proposed model achieved an accuracy of 98%. Rajasekar et al. [170] designed a hybrid learning model to identify COVID-19. They used CNN for feature extraction, and MLP was employed for classification. The model showed an accuracy of 94.89% compared with conventional MLP and CNN, where 86.95% and 80.77% accuracy were noted, respectively. Xie et al. [171] designed a CNN-based DL model to identify COVID-19 from other suspected ones. They used UNet and COVIDNet architecture for segmentation, whereas the ResNet50 network was deployed for classification. Sethy et al. [172] devised three approaches: VGG19 + SVM, VGG19, and LBP feature + quadratic SVM to identify COVID-19 patients. They used 13 pretrained DL models to compare the proposed approach. The average accuracy was 77.28%, whereas the maximum accuracy of 85.7% was achieved by LBP feature + quadratic SVM approach. Özyurt et al. [173] used shuffleNet CNN architecture to classify COVID-19 patients and reported an accuracy of 98.99%. Garain et al. [174] designed a three-layer DCSNN to screen for COVID-19. They developed two variants: spike train-based, and potential-based, and the performance was compared with three DL architectures. Of all, the potential-based model provided the optimal outcome with an accuracy of 99.51%. 

Elghamrawy et al. [175] implemented a COVID-19 classification model, AIMDP based on CNN architecture. They compared the model performance with five existing DL models, and the designed model achieved an accuracy of 98%. The authors also used WOA optimization techniques to select the most relevant patient sign. Sen et al. [176] used CNN to extract the features and the bi-stage feature selection method to identify the most relevant feature for identifying COVID-19. Finally, the SVM classification algorithm reported 90% and 98.39% accuracy for two datasets. Teodoro et al. [177] applied pretrained CNNs with three classification algorithms: KNN, SVM, and DNN. Among all, CNN EfficientNetB0 performed best along with the SVM-RBF kernel. They reported that the proposed approach achieved an average performance of 98.56%. Yasar et al. [178] used 24-layer CNN architecture with and without local binary pattern CT images for COVID-19 vs. normal. They reported that the maximum efficiency of 94.56% was obtained using no pipeline approaches instead of pipeline approaches. Brahim et al. [179] proposed a DL model named COV-CAF, and the performance was compared with four preexisting COVID-19 classification models and reported an accuracy of 97.59%. Afshar et al. [180] introduced a new COVID-19 dataset named COVID-CT-MD and applied DL and ML algorithms to check the effectiveness of the dataset. They reported that 93% accuracy was obtained by introducing the dataset and underlying studies. Liu et al. [181] specially developed an automated classification model, COVIDNet, to distinguish between COVID-19 and seven other types of pneumonia. They reported that the model achieved an accuracy of 94.3%. Kundu et al. [182] devised an ensemble model based on three CNN architectures: VGG11, wideResNet50-2, and InceptionV3. They reported an average accuracy of 98.86%, which was better than other DL architecture. Pal et al. [183] used two CNN architectures: VGG16 and InceptionV3, to classify COVID-19. They reported an accuracy of 84%, which was achieved using individual CNN models. 

Biswas et al. [184] initially used three CNN architectures: VGG16, ResNet50, and Xception. The authors introduced a stacked model (VGG16 + ResNet50+ Xception) via an ensemble learning technique, and 98.79% accuracy was reported. Helwan et al. [185] used three DL models, namely ResNet18, ResNet50, and DenseNet201. Of all, DenseNet201 performed best, with an accuracy of 98.7%. Castiglione et al. [186] proposed the ADECO-CNN approach and compared it with pretrained CNN models, namely VGG19, GoogleNet, and ResNet. They reported that the proposed approach-based model achieved an accuracy of 99.99%. Yan et al. [187] performed a quantitative analysis and designed a DL model named CovidSegNet to segment COVID-19 infections. The model performance was compared with the preexisting FCN, UNet, VNet, and UNet++ networks. Finally, the CovidSegNet model provided the best performance with a dice coefficient of 72.6% for COVID-19 segmentation. Suri et al. [188] presented a COVLIAS 1.0 system that consists of two hybrid DLs for COVID-19 segmentation. They compared the model performance with the conventional NIH model. 

Nair et al. [189] proposed the CoRNet DL model and compared it with five existing DL models: AlexNet, VGG16, SqueezeNet, VGG19, and ResNet50. CoRNet achieved high performance with an AUC value of 95%. Wan et al. [190] designed a modified AlexNet architecture and compared it with LBP + SVM, and deep feature + SVM, where AlexNet performed well with an accuracy of 94.75%. Guo et al. [191] proposed a model based on a modified version of ResNet18 to diagnose COVID-19. They reported 98.88% and 99.80% model accuracy for two- and fivefold cv. Xia et al. [192] proposed a rapid screening classifier to diagnose COVID-19. The classifier provided the best outcome with CXR and clinical features, whereas CT-based diagnosis outperformed severe cases of COVID-19. Polat et al. [193] used a CNN to identify COVID-19 and all balanced datasets. The accuracy of the proposed model was 93.26%. Li et al. [194] developed VGG16 deep learning model to classify COVID-19 vs. CP vs. NC. They reported an accuracy of 93.57%, which was achieved using a newly designed model. Owais et al. [195] proposed the DAL-Net model and compared the model performance with seven DL models: VGG16, VGG19, UNet, FCN, DeepLabV3+, MobileNetV2, and ResNet. They reported an AUC of 97.80%, which was better than others. Jia et al. [196] proposed a modified ResNet to classify COVID-19 vs. non-COVID-19 infections vs. normal control. Five CNN architectures (VGG, Inception, DenseNet, SqueezeNet, MobileNet) and two specific detection models (COVID net and CovidNet-CT) were used for comparative studies. They reported that the proposed model achieved an accuracy of 99.3%. He et al. [197] proposed multitasking multi-instance UNet to identify the severity assessment of COVID-19 and the segment of the lung lobe. They reported an accuracy of 98.5% for the assessment of COVID-19 severity. Murugan et al. [198] applied a whale optimization algorithm to ResNet50 to optimize DL architecture and built a WOANet model to classify COVID-19. The proposed architecture achieved an accuracy of 98.78%, providing a better outcome than the nonoptimized ResNet50. Naeem et al. [199] introduced a new DL model named CNN-LSTM and compared it with conventional DL models such as VGG16 and VGG19. The proposed model achieved an average accuracy of 90.98. Kalane et al. [200] proposed a UNet architecture to classify COVID-19; overall, 94.10% accuracy was reported. Fouladi et al. [201] used ResNet50, VGG16, CNN, CAENN, and machine learning approaches (NN, SVM, RF, SGD LR, and MLP) to classify COVID-19 where NN achieved high performance with an accuracy of 94%. On the other hand, the classification accuracies of ResNet50, VGG16, CNN, and CAENN were obtained as 92.24%, 94.07%, 93.84%, and 93.04%, respectively. Wang et al. [202] developed a new approach based on a deep feature fusion combination of an improved CNN model. This model performed better than the other 15 DL models, with an average accuracy of 96.66% reported. Yu et al. [203] proposed three models, ResNet101-C, NNet-C, and ResGNet-C, to classify pneumonia caused by COVID-19 vs. normal. The ResGNet-C model provided better performance with an accuracy of 96.62%. Gao et al. [204] proposed DCN for COVID-19 diagnosis that can be achieved from an individual classification level. They used internal and external datasets to evaluate this proposed model by comparing five DL models. The proposed model outperforms by attaining 96.74% and 92.87% accuracy for internal and external datasets, respectively. Sahoo et al. [205] implemented the COVIDCon model and compared it with other state-of-the-art algorithms. They reported that the proposed model attained an accuracy of 99.06% with the CT scan dataset. Lacerda et al. [206] built an AI model based on optimized VGG16 and compared it with the baseline model of VGG16. They reported that the optimized model attained an accuracy of 88%, whereas 87% accuracy was reported for the baseline model. 

Siddiqui et al. [207] introduced the ID2S-COVID19-DL system to classify COVID-19, and 98.11% system accuracy was reported. Haikel et al. [208] produced a DL model named EfficienNet-B3-GAP-ensemble and applied it to two datasets. They reported that the proposed model achieved an accuracy of 99.72% and 88.18%, respectively. Bekhet et al. [209] proposed a fully automated hybrid CNN model to classify COVID-19. They reported that the proposed model attained accuracy of 92.02%. Kaushik et al. [210] developed the VGG16 model and compared the proposed model performance with three DL models: CNN, DenseNet, and XceptionNet. The authors reported that the VGG16 model outperformed with an accuracy of 95.26%. El-Shafai et al. [211] built an automated COVID-19 detection model named SR-GAN and compared the model accuracy of 99.05% with 13 DL models. Masud et al. [212] proposed a CNN model and compared it with three DL architectures: MobileNetV2, InceptionV3, and Xception. They reported that the proposed model achieved an accuracy of 96%. El-Shafai et al. [213] used CNN architecture and studied optimizers with different batch sizes and constant learning rates. Finally, a comparative study was presented using optimizer and activation functions. They reported that the proposed model achieved 100% accuracy. 

Kassania et al. [214] proposed a method based on DenseNet121 + Bagging, and 99% accuracy was achieved by this method for the detection of COVID-19. Wang et al. [215] proposed a model based on f 3D UNet++–ResNet50 architecture for the classification and segmentation of COVID-19. They reported that the model attained an AUC score of 99.1%. Ahuja et al. [216] implemented a DL model based on ResNet18 architecture that attained 99.4% accuracy. Pu et al. [217] used the UNet network and BER algorithm to identify COVID-19 severity and progression. They reported that the proposed model performed well, with a sensitivity of 95%. Maghdid et al. [218] developed a model based on AlexNet architecture with an accuracy of 94.1%. Kumar et al. [219] used a deep neural network to detect COVID-19, and 98.4% accuracy was reported. Wang et al. [220] applied a modified Inception transfer learning model with 1065 positive COVID-19 cases. The model attained an accuracy of 79.03% on the external testing dataset.

In 2021, we found 116 articles and 36 that worked based on the private datasets to identify COVID-19. Most authors utilized CNN, ResNet-XX, VGG-XX, DenseNet-XX, and UNet architecture, and some authors introduced new models such as FewShot, FractalCovNet, CCSHNet, COVIDCon, and CovH2SD. Later, the authors applied an optimization approach to their proposed model and compared their model performance with existing models. All details are documented in Table 3. 

#### 4.3.3. 2022

Khurana and Soni [221] used four DL architectures, namely ResNet50, efficient netB0, VGG16, and CNN, to detect the presence of COVID-19. Of all, ResNet50 obtained the highest accuracy of 98.9%. Canayaz et al. [222] proposed two new methods to diagnose COVID-19 using DL and ML algorithms. Two DL models, ResNet50 and MobileNetV2, are used for feature extraction along with two classification algorithms, SVM and KNN. The total experiment was performed in three steps using individual and mixed datasets. The reported accuracies are 95.79%, 99.06%, and 99.37% for MobileNet, ResNet50 + SVM, and ResNet50 + KNN, respectively. Subhalakshmi et al. [223] proposed a DLMMF model to identify COVID-19. The proposed architecture is based on InceptionV4 and VGGNet16, which are used to extract features from the dataset. The Gaussian naïve Bayes classifier was deployed as a final classifier for disease detection. Zouch et al. [224] used two DL architectures, ResNet50 and VGG19, to detect COVID-19. Both models obtained an accuracy of 99.35% and 96.77%. Balaha et al. [225] introduced a DL framework for early detection and prognosis of COVID-19. Seven different CNNs architectures are used, and for classification, maximum accuracy of 99.61% was obtained using EfficientNetB7. The authors also reported an accuracies of 98.70% and 97.40% obtained by ensemble bagged trees and trees (fine, medium, and coarse) for the early prognostic phase. 

Habib et al. [226] proposed a classification system for COVID-19 with a hybrid feature extraction approach. Three different architectures, ResNet101, DenseNet201, and weber local descriptor, were used to classify COVID-19, lung opacity, healthy, and viral pneumonia. They reported that the proposed model achieved an accuracy of 99.3%. Montalbo et al. [227] used six DL architectures, InceptionV3, Xception, ResNet50V2, DenseNet121, and EfficientNetB0, to classify COVID-19. They compare the performance between truncated models and general models. Of all, the maximum accuracy of 97.41% was obtained using InceptionResNetV2 with truncated models. Ali et al. [228] devised a model to identify COVID-19 severity using CNN and KNN. They compared the result with the existing classification model, and 95.65% accuracy was reported. In the next experiment, modified CNN achieved an accuracy of 92.80% for detecting pneumonia on mixed data. Pandey et al. [229] proposed an efficient model to diagnose COVID-19 using three DL architectures (ResNet50, MobileNet, VGG16). The authors used image segmentation and compared the model performance. The maximum accuracies of 99.28% and 83.18% were achieved via VGG16 along with OTSU segmentation and without segmentation. 

Liu et al. [230] introduced a new framework named DCNN + IMPA (internet protocol marine predator) to diagnose COVID-19. They reported that the model achieved an accuracy of 97.57%. Luo et al. [231] developed a model to detect COVID-19 vs. normal vs. CAP using Resenet-50 and UNet. They reported a maximum efficacy of 93.84%, and 92.86% was achieved in testing and validation set via UNet. Saheb et al. [232] proposed an ADL-CDF architecture to detect COVID-19. A maximum accuracy of 98.49% was reported. Batra et al. [233] proposed a model based on the architecture of InceptionV3, and the reported accuracy was 93%. The authors also worked on X-ray images where the same model performed best. The model performance was compared with two other models, VGG16 and ResNet50V2. Cao et al. [234] introduced a CNN model to detect COVID-19, and 82.7% accuracy was achieved. They compared the model performance with three other CNNs: Goolenet-RI, ResNet50-RI, and GoogleNet-TL. Of all, the top F1 score of 79.1% was obtained via the proposed model. Yazdani et al. [235] developed a model based on CNN and NN to detect COVID-19 using low-level and deep features. Local neighborhood difference pattern was performed to extract handcrafted features, and MobileNetV2 was used to extract deep features. The optimal accuracy of 99.61% was obtained by combining texture and deep features using CNN architecture. Bhuyan et al. [139] experimented with classifying COVID-19 with CNN architecture. The authors compared the model performance with mass segmentation and without mass segmentation with a fourfold validation technique. They reported that average accuracy of 99% and 97.75% was achieved with mass segmentation and without mass segmentation, respectively. Ibrahim et al. [236] used hybrid deep learning techniques to identify COVID-19. They used three DL architectures, namely, VGGNet, CNN, high-resolution network with segmented images, and 95% accuracy was reported. Akinyelu et al. [237] performed a comparative study with 12 DL architectures: VGG16, VGG19, ResNet50, InceptionV3, Xception, MobileNetV2, ResNet101V2, DenseNet169, DenseNet121, InceptionResNetV2, NASNetLarge, and densenet201. NASNetLarge, InceptionResNetV2, and DenseNet169 provided good accuracies of 99.86%, 99.78%, and 99.71%. The authors also reported that VGG16 and densenet121 produced the highest sensitivity of 99.94%. Florescu et al. [238] proposed a model based on VGG16 with a federated learning approach to detect COVID-19. They reported that the model performed well in the training and validation phase with categorical accuracy of 83.82% and 79.32%, respectively. 

Jingxin et al. [150] introduced the DL model for COVID-19 lesion detection and segmentation. They used ResNet50 architecture, and 98.39% accuracy was reported. Baghdadi et al. [239] devised a model for COVID-19 detection on both two and three classes. Maximum accuracies of 99.74% and 98% were attained via MobileNetV3Large (two-class) and SENet154 (three-class), respectively. They also compared the model output with other CNN models like LeNet5 CNN, covid faster R-CNN, lightCNN, fuzzy + CNN, dynamic CNN, and optimized CNN. Shaik et al. [240] used various pretrained models such as VGG16, VGG19, InceptionV3, ResNet50, ResNet50V2, InceptionResNetV2, Xception, and MobileNet. Further, the authors created a strong ensemble approach using these trained models to detect COVID-19 infection. The maximum average accuracy of 93.33% was reported with 5- and 8-clf, respectively. Reis et al. [241] devised a new COVID-DSNet model to detect COVID-19 along multiclass target labels. The maximum accuracy of 97.60% was achieved via CT scans where the target labels are COVID-19 vs. normal. The author further used mixed datasets (X-ray and CT) and proposed three models: COVID-DSNet + LSTM, COVID-DSNet +FCC, and COVID-DSNet. The reported average accuracy was 95.64%. Garg et al. [242] devised a DL model based on efficient net-B5 to detect COVID-19. The model attained an accuracy of 98.45%, and 97.69% accuracy was reported for multiclass datasets. Fan et al. [243] developed a COVID-19 detection model based on CNN (ResNet152) architecture and transformer network (Deit-B). The proposed model attained a maximum accuracy of 96.7%, better than a typical CNN (95.2%). 

A 3D CNN is interesting. Karthik et al. [244] developed a DL framework based on 3D CNN, and the model performed best. Verma et al. [245] used NNs to train the model to make a CovCT application for the detection of COVID-19. The developed model attained an accuracy of 99.58%. Smadi et al. [246] developed a model named SEL-COVIDNET for the diagnosis of COVID-19, which was tuned with DenseNet121, InceptionResNetV2, and MobileNetV3Large. The authors experimented with multiclass and binary classification. They reported that their model obtained an accuracy of 98.79% (COVID-19 vs. normal). Further, the model achieved an accuracy of 98.52% for X-ray and CT mixed data. Fallahpoor et al. [247] used seven DL architectures, DenseNet169, DenseNet201, ResNet152, ResNet50, ResNeXt50, SEResNet152, SEResNeXt50, to identify COVID-19. The maximum accuracy of 82.3% was attained via ResNet50. 

Sadik et al. [248] et al. developed a model named P-DenseCOVNet, which can used in two-class (COVID-19 vs. non-COVID-19) and three-class (COVID-19 vs. pneumonia vs. healthy). They reported 93.8% and 87.5% accuracy obtained on two- and three-class COVID-19 detection, respectively. Huang et al. [249] proposed the LightEfficientNetV2 model and compared the performance with the two best other models, namely MobileNetV2 (without tuning) and Xception (with tuning). They reported that 97.48% accuracy was attained via LightEfficientNetV2, which was best compared to two other models. Li et al. [250] proposed a MultiR-Net, a 3D deep learning model to classify COVID-19 and lesion segmentation. The proposed model performance was compared with four different models: DenseNet, Res2Net, Zhou’s, and JCS. They reported that the highest classification accuracy of 92.647% was achieved using MultiR-Net. Hemalatha et al. [251] used a hybrid random forest deep learning classifier to detect COVID-19, and 99% accuracy was reported. The authors also claimed that their proposed methodology is fitted for edge computing with higher detection accuracy. Wang et al. [252] built an SSA-Net segmentation model, which helps to diagnose COVID-19, and 70.31% DSC was reported. Qi et al. [253] used a capsule network with ResNet50 for slice-level prediction, and 93.4% accuracy was reported. Also, the authors claimed their method achieved 100% accuracy for patient-level prediction. 

Oğuz and Yağanoğlu [254] proposed a hybrid method combining in-depth features extracted from ResNet50, and SVM was used as a final classifier to detect COVID-19. They used AlexNet, ResNet50, ResNet101, VGG16, VGG19, GoogleNet, SqueezeNet, and Xception architecture to extract deep features. Five classification algorithms, SVM, RF, KNN, DT, and NB, were deployed on extracted features. Finally, maximum accuracy of 96.296% was obtained via the SVM classifier with ResNet50. Ravi et al. [111] used EfficientNet architecture to predict COVID-19, and 99% accuracy was reported. The model performance was compared with others pretrained. The authors also used the t-SNE method to visualize CT test data. Yang et al. [255] proposed the F-EDNC model to recognize COVID-19 and compared the performance with FC-EDNC, O-EDNC, and CANet. The maximum efficacy of 97.55% was attained via F-EDNC. Mijares et al. [256] used CNN to diagnose COVID-19, and 94.89% classification accuracy was reached. Heidari et al. [257] utilized blockchain-based CNNs to detect COVID-19. They reported that 99.34% and 99.76% accuracy was attained in the testing phase for four- and two-class classifications. Singh and Kolekar [115] developed a fine tune model based on MobileNetV2 architecture to diagnose COVID-19. The model performance was compared with three deep learning models such as VGG19, DenseNet201, and VGG16. They reported that the proposed model attained an efficacy of 96.40% with ten-times shorter response time. Ortiz et al. [258] devised a prognosis model for COVID-19, and 91% accuracy was reported. The authors noted that the accuracy label was improved in CT features, patient demographics, and image segmentation. Sangeetha et al. [259] deployed two DL architectures, VGG19 and ResNet152V2, to diagnose COVID-19. They reported that both models achieved an accuracy of 98%. Mohammed et al. [260] proposed an optimal deep learning model based on ResNet50 architecture to diagnose COVID-19. A total of 15 DL models were used to compare the performance, and a maximum of 91.46% accuracy was reached via ResNet50. In contrast, InceptionV3 provided the lowest performance.

Joshi et al. [261] introduced the MFL-Net model to recognize COVID-19 using individual and combined datasets. They reported an average accuracy of 96.12%, whereas 96.13% was attained via the mixed form. Zhang et al. [262] introduced a DL model based on VGG19 with globalmaxpool2D to detect COVID-19. They reported that the proposed model achieved an accuracy of 94.12%, which was best compared to others. Mouhafid et al. [263] utilized two ensemble learning methods, stacking and weighted average ensemble (WAE), to combine the performance of three fine-tuned-based learners such as VGG19, ResNet50, DenseNet201. The result showed that the maximum accuracy of 98.59% and 95.05% was achieved via WAE. Dara et al. [264] applied ResNet architecture to implement the classification algorithms to identify COVID-19. The authors used ResNet18, ResNet50, ResNet101 and compared their performance. Finally, ResNet39 was chosen and used the parameters obtained from ResNet18, ResNet50, ResNet101. The global and local models achieved an accuracy of 97.53 and 90.66, respectively. Ozdemir et al. [265] used ResNet50 architecture and extended it with a feature-wise attention layer to classify COVID-19. They reported that 95.57% accuracy was obtained via the proposed model. Ahuja et al. [266] devised a COVID-19 classification model named McS-Net based on ResNet18, and 98.07% accuracy was reported. Messaoud et al. [267] used VGG19 architecture to identify COVID-19, and model performance was compared with efficient net-B4 + CLAHE. They reported that the proposed model achieved an accuracy of 86%. The authors also worked on X-ray and combined datasets where the model attained accuracies of 97% and 90%, respectively. 

As before, Manconi et al. [268] proposed a 3D inception CNN architecture to detect COVID-19. Especially, 3D InceptionV1 and InceptionV3 models were built and compared their performance. Further, an ensemble classifier is deployed on CNN models. The maximum accuracy of 98.21% was reached via InceptionV1 with a voting strategy. Cheng et al. [269] designed a COVID-19 detection model based on VBNet + LSTM, and 89% accuracy was reported. Lu et al. [270] introduced a new COVID-19 detection system named CGNet. The model architecture is based on the combination of ResNet18 and k nearest neighbors. The proposed model achieved an accuracy of 97.78%, and Grad-CAM provided visual explanation. Owais et al. [271] proposed DSS-Net to identify COVID-19 and 96.58% accuracy was reported. Yoo et al. [272] developed the 2D UNet model to classify the COVID-19 disease. The authors compared their model performance along with internal and external validation. The Pearson correlation coefficient suggested that the model performs well between UNet outputs and visual CT scores. Suri et al. [273] proposed a model combining DL and hybrid DL to track lesion location and segmentation. They used VGG-SegNet, ResNet-SegNet, VGG-UNet, and ResNet-UNet. The best AI model was ResNet-UNet, with a 92% correlation coefficient with a prediction time of less than 1 s. Ghose et al. [274] proposed a DL model based on densenet-169 to recognize COVID-19. They reported that the proposed model achieved an accuracy of 99.95%. The authors also worked on X-ray images, and 99.59% accuracy was reached. 

Gunraj et al. [275] proposed Covid-Net CT architecture to detect COVID-19. A maximum accuracy of 99% is reported. Yousefzadeh et al. [276] used UNet architecture for lobe segmentation, and the KNN classifier was applied to predict the severity of infection due to COVID-19. They reported that the proposed model has a 71% to 74% dice score. Choudhary et al. [277] experimented on two DL models, VGG16 and ResNet34, to detect COVID-19. The optimal accuracy of 95.47% was obtained via resnet34. Chouat et al. [278] used four DL architectures, namely VGGNet19, ResNet50, InceptionV3, and Xception, to detect COVID-19. Of all, VGGNet19 and Xception models outperformed with an accuracy of 90.5% and 89.5%, respectively. Dialameh et al. [279] proposed a DL model called Deep CT-Net on DenseNet121 to detect COVID-19. They reported that the proposed model attained an AUC of 88.6%. Venkatachalam et al. [280] proposed a CNN model with BBO that helps the layers selection process. The proposed model performance was compared with existing models, namely VGG16, InceptionV3, ResNet50, MobileNet. The result showed that the proposed model outperformed InceptionV3 and ResNet50. The reported accuracy was 98.5% and 97.6% in the test and train phases, respectively. Latif et al. [281] introduced a hybrid method using ResNet18 and GoogleNet2000 and the extracted features via SVM. They reported that the proposed model achieved an accuracy of 99.91%. El-Shafai et al. [282] devised a CNN framework to detect COVID-19, and 98.49% accuracy was reported. Xue et al. [283] used CNN architecture to identify COVID-19. The proposed model achieved an accuracy of 97.67%. El-Shafai et al. [284] proposed a CNN structure to detect COVID-19, and 100% accuracy was reported. 

In 2022, we found 68 articles, and 14 articles worked on a private dataset to identify COVID-19. Most authors used CNN, ResNet-XX, VGG-XX, DenseNet-XX, and EfficientNet-XX architecture. A few authors invented hybrid frameworks like SpaSA and CNN, MOMHTS optimized hybrid random forest deep learning, and ResNet18 + GoogleNet2000 features with SVM. Further, a few authors also worked on X-ray images and combined their CT data with them. The details are documented in Table 4.

### 4.4. Performance Comparison

This section compares the performance of different DL architectures used from 2020 to 2022. There has been substantial research on COVID-19 screening utilizing chest CT scans from the beginning of 2020. A fair comparison among the authors can only be made when they have used exact data collection, evaluation protocol, and performance metrics. Unlike other healthcare issues, in COVID-19, there has been a growing tendency in dataset size resulting in incremental studies over time. As high-end machine learning methods, such as deep learning models, require a massive quantity of data, writers have explored the usage of data collection size. In our study, convolutional neural networks (CNNs) are the most popular, followed by residual neural network (ResNet). In what follows, we categorized the authors’ work using the same architecture (see Table 5, Table 6, Table 7, Table 8, Table 9, Table 10, Table 11 and Table 12).

Convolutional neural network (CNN): Eighty-seven authors utilized CNN architecture to detect COVID-19 from 2020 to 2022. Among them, 22, 48, and 17 articles were identified in 2020, 2021, and 2022, respectively (see Table 5). Finally, El-Shafai et al. [284] proposed a model in 2022 that achieved the highest accuracy of 100%.

Residual neural network (ResNet): Fifty-two authors used ResNet architecture and 12, 21, and 19 papers were documented in 2020, 2021, and 2022, respectively (see Table 6). In this architecture, fifteen authors preferred private datasets to build their model. Finally, Ahamed et al. [153] proposed a model that achieved the highest accuracy of 99.99% using the C15 dataset. 

Visual geometry group (VGG): Twenty studies were reported using this network, where 3, 15, and 9 articles were found in 2020, 2021, and 2022, respectively (see Table 7). Balaha et al.’s [16] proposed model achieved the highest accuracy of 99.33% using C1, C3, and C24 datasets. Also, three authors used private datasets.

Densely connected convolutional networks (DenseNet): Fourteen studies were identified where authors used this framework. The highest accuracy of 99% was achieved by Kassania et al. [214]. Most authors preferred publicly available datasets; only three worked on private datasets (See Table 8).

Inception: Nine authors used the Inception network for their study, and three worked on private datasets. Finally, El-Bana et al. [93] achieved the highest accuracy of 99.5% with 99.8% sensitivity.

UNet: Twenty-seven articles were identified, and 14 researchers utilized private datasets. The highest accuracy of 99% was achieved by Munusamy et al. [146].

MobileNet: Three authors used this architecture, and Canayaz et al.’s [222] proposed model achieved the highest accuracy of 99.06% using C1 and C4 datasets.

EfficientNet: Five authors preferred this network in their research, where the highest accuracy (99.61%) was obtained by Balaha et al. [225].

### 4.5. How Big Is Big Data?

The greater the data, the higher the performance we state in machine learning, particularly for DL-based models. As far as machine learning algorithms are concerned, this is not true, as it necessitates all possible manifestations associated with a specific disease (COVID-19, in our case). However, the size of the dataset allows for the likelihood of having new topics (i.e., manifestations). We refer to earlier works for additional information [285,286]. Underfitting and overfitting were not discussed in most of the published papers due to the lack of data. Furthermore, the authors used the holdout method to train/test the model instead of k-fold cross-validation. Another critical issue is an unbalanced dataset, where possible bias was not well argued.

### 4.6. Transfer Learning

Apart from conventional machine learning, large amounts of annotated data are required for DL models to deliver desirable performance. For this reason, transfer learning is usually applied and generates a large amount of data. Subsequently, the model is fine-tuned for a target task of interest. The use of pretrained DL models is prevalent in COVID-19 imaging tools. For example, commonly used models are AlexNet, VGGNet-XX, GoogleNet, ResNet-XX, Xception, Inception, wideResNet, MobileNet, NASNet, DarkNet, CheXNet, ShuffleNet, InceptionResNetV2, InceptionV3 (to name a few) [62,67,79,95,102,103,111,112,114,115,121,125,128,134,139,143,146,151,152,153,158,159,162,210,211,213,214,220,234,247,249,255,263,270,274,277,284].

### 4.7. Data Augmentation

Data augmentation plays a significant role in reducing overfitting, which can improve the performance of DL models. The principal objective of the augmentation technique is to uplift the available raw data by adding slightly modified copies of the source or, in some cases, the synthetic image generated from the existing data. For COVID-19 imaging tools, authors often used augmentation methods for aiming more features from the limited available data. Let us discuss the use of augmentation techniques on CT imaging tools over the last two years (see Table 13). 

#### 4.7.1. 2020

We found a total of 10 articles; among them, the researchers of 9 articles used conventional augmentation techniques such as resizing, zooming, gaussian noise, blur, spatial transformation, contrast adjustment, flipping, scaling, cropping, rotation, intensity transformation [69,71,75,84,85,92,93,96,145]. Similarly, one author used GAN [62], considered under the classical augmentation approach. 

#### 4.7.2. 2021

We found 16 articles where researchers applied different types of augmentation techniques. Most of the authors used resizing, rotating, flipping, skewing, cropping, color jittering, patching, flattering, downsampling, transpose rotation, squeezing, shearing, reflection, coronal view, symmetric rotation, noise injection, HS transform, vs. transform, rotation, GC, RT [105,113,114,121,147,150,153,159,163]. Similarly, a few authors utilized GAN [110,211], SRGAN [120], CycleGAN [148], and RAIOSS [139] approaches. 

#### 4.7.3. 2022

A total of 12 articles are listed where authors used data augmentation methods. The most popular approaches are horizontal flip, anticlockwise rotation, scaling, brightness change, and contrast enhancement [224,236,237,239,244,261,284]. In addition, GANs, cycle GAN, CCGAN [225], and RAIOSS [139] were also opted for in a few studies. 

## 5. Conclusions and Future Scope

In this paper, we studied peer-reviewed research findings/articles on AI-guided tools for COVID-19 analysis/screening using chest CT scans images in the years 2020, 2021, and 2022. Our research was confined to deep learning methods for detecting COVID-19 in CT scans, and we identified data collections, methodological procedures, and discussion of prospective methodologies and challenges. Using the search terms (*COVID-19* OR *Coronavirus*) AND *chest CT* AND (deep learning OR artificial intelligence OR medical imaging), we systematically reviewed 231 research papers and meta-analyses on the PubMed Central Repository and Web of Science. Unlike standard articles, we did not analyze pre-print publications like those in ArXiv, TechRxiv, and medRxiv.

Future guidelines for effectively using CT imaging and deep learning (DL) in COVID-19 screening should prioritize data augmentation (DA) and diversity to enhance DL model generalizability. Recommendations include augmenting data with varying noise levels and incorporating scans from diverse populations, disease stages, and comorbidities. Finetuning existing models on COVID-19 data helps to learn disease-specific features while leveraging knowledge from pretraining on other medical images. Emphasizing explainability and interpretability is essential, using techniques like attention maps, saliency maps, or Grad-CAM to highlight influential regions in CT scans, increasing trust in DL model decisions. 

## Figures and Tables

**Table 1:** Different CT datasets according to COVID-19-positive cases.

Sl. No	Dataset	Area of Utilization	Total Size (#Positive Cases)	Availability (accessed on 2 June 2023)
C1	Covid-CT	Classification	746 (#349)	https://arxiv.org/abs/2003.13865
C2	COVID-19 CT	Segmentation	829 (#373)	http://medicalsegmentation.com/COVID-19
C3	Large COVID-19 CT scan	Classification	12,058(#2282)	https://github.com/mr7495/COVID-CTset
C4	SARS-CoV-2 Dataset	Identification	2482 (#1252)	https://www.kaggle.com/plameneduardo/sarscov2-ctscan-dataset
C5	COVID-19 open research dataset (CORD-19)	Identification	3439 (#98)	https://www.kaggle.com/allen-institute-for-ai/CORD-19-research-challenge
C6	SIRM COVID-19 database	Classification	100(#100)	https://www.sirm.org/en/category/articles/COVID-19-database/
C7	COVID-19 BSTI imaging dataset	Classification	Not provided	https://bit.ly/BSTICovid19_Teaching_Library
C8	Radiopaedia	Classification	36,559 (#3520)	http://radiopaedia.org/articles/COVID-19-3
C9	MosMeddata	Classification	1110 (#1110)	http://mosmed.ai/datasets/COVID-19_1110
C10	COVID-19	Classification	521 (#48)	https://github.com/KevinHuRunWen/COVID-19
C11	COVID-CS	Classification and Segmentation	3855 (#200)	https://github.com/yuhuan-wu/JCS
C12	BIMCV-COVID-19	Detection	6687(#1311)	https://github.com/BIMCV-CSUSP/BIMCV-COVID-19
C13	COVID-19-CT-CXR (classification)	Classification	1327 (not provided)	https://github.com/ncbi-nlp/COVID-19-CT-CXR
C14	Larxel dataset	Segmentation	20(#20)	https://www.kaggle.com/andrewmvd/COVID-19-ct-scans
C15	Large COVID-19 CT scan	Classification	17,102 (#7593)	https://www.kaggle.com/maedemaftouni/large-COVID-19-ct-slice-dataset
C16	Extensive COVID-19 X-Ray and CT Chest Images Dataset	Classification	17,099 (5427)	https://data.mendeley.com/datasets/8h65ywd2jr/3
C17	CF data	Detection	19,685 (4001)	http://ictcf.biocuckoo.cn/HUST-19.php
C18	Covid19 Image dataset	Classification	22,873 (#3520)	https://arxiv.org/abs/2003.11597
C19	CC-CCII	Classification	4178 (#1544)	http://ncov-ai.big.ac.cn/download
C20	COVID-CT-MD	Classification	Private	https://figshare.com/s/c20215f3d42c98f09ad0
C21	Deep Covid	Classification	5000(#Not given)	https://github.com/shervinmin/DeepCovid/tree/master/
C22	CT scan for COVID-19	Classification	13,980 (#4001)	https://www.kaggle.com/azaemon/preprocessed-ct-scans-for-COVID-19
C23	Covid Chest Xray and CT images	Classification	144 (#118)	https://github.com/ieee8023/covid-chestxray-dataset
C24	Harvard Dataverse	Classification	4172 (#2167)	https://dataverse.harvard.edu/dataset.xhtml?persistentId=doi:10.7910/DVN/SZDUQX
C25	COVIDx CT	Classification	431,205 (#316774)	https://www.kaggle.com/datasets/hgunraj/covidxct

Table 1 lists 25 CT scan datasets utilized for COVID-19 screening. Out of these datasets, 19 are employed for classification purposes, 3 for segmentation tasks, and 2 for identification and detection purposes, respectively.

**Table 2:** Chest CT imaging tools, dataset, and their performance are measured in terms of accuracy (ACC), specificity (SPEC), sensitivity (SEN), and area under the curve (AUC) for 2020.

Authors [Ref]	Methods	Dataset Collection	Performance (%)			
			ACC	SPEC	SEN	AUC
Ni et al. [60]	MVP-Net and 3D UNet	Private	Per lobe—83 Per patient—94	-	96 100	86.54 86.08
Hu et al. [61]	DenseNet169	C1	86.00	-	-	94.00
Loey et al. [62]	ResNet50	C1	82.91	87.62	77.66	-
Song et al. [63]	BigBiGAN (DNN)	Private	-	91	92	97
Chaganti et al. [64]	DenseUNet	Private	-	-	-	-
Singh et al. [65]	Mode-based CNN	Private	93.5	~90	~90	-
Ning et al. [66]	CNN	C17	-	-	-	89.6
Jaiswal et al. [67]	DenseNet201	C4	96.25	96	96	-
Babukarthik et al. [68] *	GDCNN	C23	98.84	97.0	100	-
Mohammed et al. [69]	RenNext+	C19	77.6	79.3	85.5	-
Han et al. [70]	AD3D-MIL	Private	97.9	-	-	99
Jiang et al. [71]	UNet	C2	-	-	-	-
Gunraj et al. [72]	COVIDNet-CT	C3	99.1	99.9	97.3	-
Fan et al. [47]	Inf-Net Semi Inf-Net	C2	-	97.4 97.7	87.0 86.5	-
Mishra et al. [73]	Deep CNN based decision fusion	C1	86	-	-	88.3
Javor et al. [74]	ResNet50	Private	-	93.3	84.4	95.6
Silva et al. [75]	EfficientCovidNet	C1, C4	87.68	-	-	-
Pathak et al. [76] *	ResNet50	C1	93.0	91.4	94.7	-
Wu et al. [77]	ResNet50	Private	76.0	61.5	81.1	81.9
Peng et al. [78] *	DenseNet121	C13	-	-	78.0	89.1
Qian et al. [79]	2D-CNN	Private	-	97.49	98.99	99.93
Li et al. [80]	CovNet	private	-	-	-	96.0
Lessmann et al. [81] *	CO-RADS	Private	-	89.8	85.7	95
Jin et al. [82] *	ResNet152	C9, C20	94.98	95.76	90.19	97.71
Jamshidi et al. [83] *	DCNN	C1, C16, C25	98.49	-	-	-
wang et al. [84] *	DeCoVNet	Private	90.1	-	-	95.9
Zhang et al. [85]	CoVNet	Private	-	-	-	95.9
Lai et al. [86]	DCNN	Private	-	-	-	91
Liu et al. [87]	DenseNet	C1, C17	-	-	-	76.09
Panwar et al. [88] *	VGG19	C4, C25	95.61	97.22	76	-
Misztal et al. [89]	CNNs	C1, C19	-	-	-	-
Amyar et al. [90]	UNet	C1, C2	94.67	92	96	-
Polsinelli et al. [91]	CNNs	C6	85.03	81.95	87.55	-
Ko et al. [92] *	ResNet50	C6	96.97	100	99.58	-
El-Bana et al. [93] *	InceptionV3	Private	99.5	99.2	99.8	-
Wang et al. [94]	3D ResNet	Private	93.3	-	95.5	97.3
Deng et al. [95]	VGG16	C25	75	-	-	-
Hu et al. [96]	NTS-NET	Private	87.1	91.23	80.83	90.6
Li et al. [80]	CoVNet	Private	-	96	90	96
Xu et al. [97]	CNN	Private	86.7	-	-	-
Wang et al. [98]	Covid19Net	Private	85.00	79.35	71.43	90.11
Kang et al. [99]	Multiview representation learning (Vnet + NN)	private	95.5	96.6	93.2	-
Chen et al. [100]	UNet++	Private	98.85	99.16	94.34	-
Bai et al. [101]	DNN Efficient Net B4	Private	96	93.2	95	95
Zhu et al. [102]	VGG16	C25	-	-	-	-
Benbrahim et al. [103]	InceptionV3 and ResNet	Private	99.01	100	72	-
Sharma et al. [104] *	ResNet	C1, C9	91		-	-

* Other data collections were also used.

**Table 3:** Chest CT imaging tools, dataset, and their performance are measured in terms of accuracy (ACC), specificity (SPEC), sensitivity (SEN), and area under the curve (AUC) for 2021.

Authors [Ref]	Methods	Data Collections	Performance (%)			
			ACC	SPEC	SEN	AUC
Ibrahim et al. [105] *	VGG19 + CNN	C6	98.05	99.5	-	99.66
Goncharov et al. [106] *	Multitask spatial-1	C2	-	-	-	0.97 ± 0.01
Zhang et al. [107]	5L-DCNN-SP-C	Private	93.64 ± 1.42	94.00 ± 1.56	93.28 ± 1.50	-
Song et al. [108]	DRE-Net (ResNet50)	Private	86	77	-	95
Yao et al. [109]	CNN	Private	-	90.5	91.5	95.5
Acar et al. [110]	CNNs	C2, C1	-	-	-	-
Ravi et al. [111] *	EfficientNet-CNN	C16	99	-	-	-
Chen et al. [112] *	ResNet50	private	91.21	88.46	94.87	-
Huang et al. [113] *	FaNet	Private	98.28	-	-	-
Jangam et al. [114] *	VGG19 + DenseNet169	C1, C3, C4	91.49	-	-	-
Singh et al. [115]	MobileNetV2	C4	96.40	-	98	99.5
Alirr et al. [116]	UNet (FCN)	C2, C6	-	95.1	82.2	-
Kundu et al. [117]	Bagging of VNNs (ET-NET)	C4	97.81	97.77	97.81	-
Saad et al. [118]	CNN (DFC)	C16	98.9	-	-	-
Fung et al. [119]	SSInfNet	C2	-	-	-	98.66
Tan et al. [120]	SRGAN +VGG16	C1	98	94.9	99	-
Lascu et al. [121] *	ResNet101	C1	94.9	-	-	-
Lassau et al. [122]	NN	Private	-	-	-	-
Pan et al. [123]	CNN	Private	-	-	-	-
Yan et al. [124]	ResNet50	C4	96.3	-	-	-
Shalbaf et al. [125]	The majority voting of five deep transfer learning architecture (EfficientNetB0, EfficientNetB3, EfficientNetB5, InceptionResNetV2, Xception)	C1	85	-	85	-
Rahimzadeh et al. [126]	ResNet50V2	C3	98.49	-	94.96	-
Lee et al. [127]	CNN	C5	-	-	-	80
Mishra et al. [128] *	CNN	C1	99	-	-	98.6
Zhang et al. [129]	ResAUNet	Private	-	-	-	-
Barbosa et al. [130] *	CNN	Private	-	-	-	
Zhao et al. [131]	SP-V-Net	Private	94.60	92.70	96.70	94.70
Jadhav et al. [132]	Covid-View	Private	95.2	94.9	95.3	98.5
Guiot et al. [133] *	VGG16	Private	85.18	91.63	69.52	88.2
Yao et al. [134]	CSGBBNet	C1, C5	98.49	97.95	99.0	-
Singh et al. [135]	Ps-ProtoPNet	C20	99.29	-	-	-
Zhu et al. [136] *	ResNet50	C1	93	92	93	-
Kuchana et al. [137]	UNet	C14, C2	-	-	-	-
Khalifa et al. [138]	CNN	C8, C14	99.3	95	98.12	-
Bhuyan et al. [139] *	FrCN (CNN)	C2	99	99.41	96.66	-
Heidarian et al. [140]	COVID-FACT	C22	90.82	86.04	94.55	98.0
Ahsan et al. [141] *	MobileNetV2	Private	98.5	-	-	81.6
Zhang et al. [142]	GARCD	C4	-	91.16	96.97	98.7
Chaddad et al. [143] *	Deep CNNs	Private	82.80			88.16
Yousefzadeh et al. [144] *	Ai-Corona (CNN)	C20, C9,	-	92.7	94.5	95.6
Chen et al. [145]	ResNet50	C1, C6, C2	86.8	-	-	93.1
Munusamy et al. [146]	FractalCovNet	C2	99	-	-	-
Wang et al. [147] *	CCSHNet	Private	-	-	96.25	-
Jiang et al. [148]	CNNs	C1	96	-	-	98.89
Hu et al. [149]	DSN-SAAL	C1, C3, C2	95.43	-	-	-
Jingxin et al. [150] *	Ours-SP (ResNet50)	C1, C19	97.83	-	96.89	-
Balaha et al. [151]	CovH2SD (VGG19)	C1, C3, C24	99.33	-	-	-
Turkoglu et al. [152]	MKs-ELM-DNN	C1	98.36	98.44	98.28	98.36
Ahamed et al. [153] *	ResNet50V2	C15	99.99	-	-	-
Pathan et al. [154]	CNN	C1	96	96	97	-
Cruz et al. [155]	CNNs	C1	86.70	-	89.52	90.82
Hasan et al. [156] *	Deep CNN	C4, C1	90.14	88.59	-	94.60
Basset et al. [157] *	U -Net	C22	96.80	-	-	98.86
Fu et al. [158]	DenseANet	C3	90.27	88.77	92.26	95.64
Aslan et al. [159] *	mAlexNet-BiLSTM (CNN)	C19	98.70	-	-	99
Kundu et al. [160]	CNNs	C3	98.93	98.93	98.93	-
Müller et al. [161] *	3D UNet	C25	-	-	-	-
Li et al. [162]	CheXNet	C1	87	-	-	75
Zhang et al. [163]	MIDCAN	Private	98.02	97.95	98.10	-
Xu et al. [164] *	Semi-CARes-UNet	C2	96.1	-	78.6	-
Mondal et al. [165]	CO-IRv2	C2, C4	96.18	97.96	-	-
Chen et al. [166]	Covid-CNN	C10	96.7	-	95.6	-
Alshazly et al. [167]	Deep CNNs	C1, C4	96.15	96.75	95.9	-
Voulodimos et al. [168] *	Few-shot UNet	C2, C8	-	-	-	-
Khan et al. [169]	MC-SVM + AlexNet + VGG16	C8	98	-	-	99
Rajasekar et al. [170]	CNN + MLP	C3	94.89	95	-	-
Xie et al. [171] *	CNN	Private	-	80.0	83.6	90.6
Sethy et al. [172] *	VGG19	Private	64.80	-	-	-
Özyurt et al. [173] *	ShuffleNet	C25	99.98	-	-	-
Garain et al. [174]	DCSNN	C1	99.51	100	98.96	-
Elghamrawy et al. [175]	CNNs	Private	98	-	98.8	96
Sen et al. [176]	CNN	C4, C1	94.19	-	-	95.5
Teodoro et al. [177]	EfficientNetB0	C4	-	98.53	98.53	-
Yasar et al. [178]	CNN	C1, C4, C3	95.16	94.01	97.54	99.06
Brahim et al. [179]	COV-CAF	C9	97.67	98.41	97.57	-
Afshar et al. [180]	DNN	Private	93	-	-	-
Liu et al. [181] *	COVIDNet	Private	94.3	88.50	91.12	98
Kundu et al. [182]	CNNs	C4, C26	98.86	98.86	98.87	-
Pal et al. [183] *	CNN	C1	84	-	-	-
Biswas et al. [184]	Stacked model (VGG16 + Xception + ResNet50)	C4	98.79	-	-	98.80
Helwan et al. [185]	DCNN	Private	98.7	97.3	98.1	-
Castiglione et al. [186]	ADECO-CNN	C4	99.99	99.97	99.92	-
Yan et al. [187]	COVID-SegNet	Private	-	-	75.1	-
Suri et al. [188]	COVLIAS 1.0	Private	-	-	-	96.75
Nair et al. [189]	CorNet	C4	-	96	90	95
Wan et al. [190]	Modified AlexNet	Private	94.75	96.69	93.22	-
Guo et al. [191]	Modified ResNet	Private	99.34	-	-	-
Xia et al. [192]	DNN	Private	96.15	81.2	94.2	-
Polat et al. [193] *	CNN	C6	93.26	93.24	92.37	-
Li et al. [194]	VGG19	C20	93.57	94.21	93.93	-
Owais [195] *	DAL-Net	C2, C9	-	85.68	99.4	97.80
Jia et al. [196] *	Modified ResNet	C25	99.30	-	-	-
He et al. [197]	M^2^U-Net	Private	98.50	-	-	99.1
Murugan et al. [198]	Optimized ResNet50	C4	98.78	99.19	98.37	-
Naeem et al. [199] *	CNN + LSTM	C4, C6	90.98	-	-	-
Kalane et al. [200]	UNet	C1, C6	94.10	93.47	94.86	-
Fouladi et al. [201]	CNNs	C4	93.3	-	87.67	-
Wang et al. [202]	FGCNet	Private	96.66	-	-	-
Yu et al. [203]	ResGNet-C	Private	96.62	95.91	97.33	-
Gao et al. [204]	DCN	Private	94.80	94.45	95.42	98.17
Sahoo et al. [205] *	COVIDCon	C20	99.06	-	-	-
Lacerda et al. [206] *	VGG16	C9	88	-	97	-
Siddiqui et al. [207]	CNN	Private	95.54	97.06	94.38	-
Haikel et al. [208]	EfficienNet-B3- GAP-ensemble	C4, C1	99.72	-	99.80	99.99
Bekhet et al. [209] *	CNNs	Private	92.08	-	-	-
Kaushik et al. [210]	VGG16	C4	95.26	95.10	95.30	-
El-Shafai et al. [211]	SR-GAN + TCNN	C1, C25, C16	99.05	-	-	-
Masud et al. [212]	CNN	C16	96	-	-	99
El-Shafai et al. [213]	CNN	C16	100	-	-	-
Kassania et al. [214] *	DesNet +Bagging	C25	99.0	99.0	99.0	-
Wang et al. [215]	3DUNet++–ResNet50	Private	-	99.2	97.4	99.1
Ahuja et al. [216] *	ResNet18	C1	99.4	98.6	100	99.65
Pu et al. [217]	UNet BER algorithm	Private	-	84	95	-
Maghdid et al. [218]	AlexNet (TL)	526	94.1	100	72	-
Kumar et al. [219]	DNN	C1	98.4	98.3	98.5	-
Wang et al. [220]	Modified Inception TL	Private	79.30	83	67	-

* Other data collections were also used.

**Table 4:** Chest CT imaging tools, dataset, and their performance are measured in terms of accuracy (ACC), specificity (SPEC), sensitivity (SEN), and area under the curve (AUC) for 2022.

Authors [Ref]	Methods	Data Collection	Performance (in %)			
			ACC	SEN (rec)	SPEC	AUC
Khurana and Soni [221] *	ResNet50	C23	98.9	98.6	99.2	-
Canayaz et al. [222]	ResNet and MobileNet, SVM-KNN	C1 and C4	95.79, 99.06,99.37	95.83	95.75	-
Subhalakshmi et al. [223]	VGGNet16, InceptionV4 + Gaussian naïve Bayes	C1	96.81	96.53	95.81	-
Zouch et al. [224] *	ResNet50 and VGG19	C1	98.06	-	-	-
Balaha et al. [225]	EfficientNetB7	C18	99.61	99.62	-	99.98
Habib et al. [226] *	ResNet101 + DenseNet201 + WLD	Not provided	99.3	99.1	-	-
Montalbo et al. [227] *	InceptionResNetV2-Tr	C22, C2, C9, C1, C18	97.41	97.52	-	99.0
Ali et al. [228] *	Modified CNN	C18	92.80	-	-	-
Pandey et al. [229]	VGG16	C15	99.28	-	-	-
Liu et al. [230]	DCNN-IPMPA	C1, C4	97.21 and 97.94	96.21 and 95.22	95.76 and 95.43	-
Luo et al. [231]	UNet	Private	93.84	93.15	-	-
Saheb et al. [232]	CNN	C3	98.49	96.83	96.83	-
Batra et al. [233]	InceptionV3	C1	93	89.81	-	-
Cao et al. [234]	ResNet50	Private	82.7	79.1	-	-
Yazdani et al. [235]	CTFDF	Private	91.61	-	-	-
Bhuyan et al. [139] *	CNN	C1	99	95.82	99.26	-
Ibrahim et al. [236]	VGGNet + CDBN + HRNet	C1	95	95	96	-
Akinyelu et al. [237]	NASNetLarge	Private	99.86	99.83	99.90	-
Florescu et al. [238]	VGG16FL	Private	1.57	-	-	-
Jingxin et al. [150]	ResNet50	C8, C18, C1	98.39	-	-	-
Baghdadi et al. [239] *	Hybrid (SpaSA and CNN)	C15	99.73	-	-	-
Shaik et al. [240]	CNNs	C1, C4	93.33	93.25	-	93.25
Reis et al. [241]	CNNs	C4	97.60	100	-	-
Garg et al. [242]	EfficientNetB5	C4	98.45	96.82	98.83	-
Fan et al. [243]	Trans-CNNNet	C25	96.73	97.76	96.01	-
Karthik et al. [244]	3D CNN	C9	-	-	-	-
Verma et al. [245]	EffecientNetB0		99.58	99.69	-	-
Smadi et al. [246]	CNNs	C4	98.79	98.8	98.8	-
Fallahpoor et al. [247]	ResNet50	Private	85%	-	-	-
Sadik et al. [248]	P-DenseCOVNet	C19	93.8	97.5	90.0	-
Huang et al. [249]	LightEfficientNetV2	C1, C4	97.48	-	-	-
Li et al. [250]	CNN	Private	92.647	93.323	-	-
Hemalatha et al. [251]	MOMHTS optimized hybrid random forest deep learning	C1	99	99	-	-
Wang et al. [252] *	ResNet34	C2	-	-	-	-
Qi et al. [253]	ResNet50	C19	93.4	-	-	87.6
Oğuz and Yağanoğlu [254]	ResNet50 + SVM	Private	96.29	95.082	-	98.21
Ravi et al. [111]	EfficientNet	C16	99.00	99.00	-	-
Yang et al. [255]	CNN	C4	97.55	96.41	98.14	-
Mijares et al. [256]	CNN	C4	94.89	90.43	-	-
Heidari et al. [257]	CNN	private	99.76	99.40	-	-
Singh and Kolekar [115]	MobileNetV2	C4	96	98	-	-
Ortiz et al. [258] *	InceptionResNetV2	C19	91	33	-	80.0
Sangeetha et al. [259]	VGG19 and ResNet152V2	C15	98	-	-	-
Mohammed et al. [260]	ResNet50	C1	91.46	-	-	-
Joshi et al. [261]	CNN	C1, and C4	96.13	-	-	96.13
Zhang et al. [262]	VGG19	Private	94.12	91.40	96.95	97.44
Mouhafid et al. [263]	WAE	C4, C1	96.82	97.25	-	-
Dara et al. [264]	ResNet39	C1, C2, C9	97.53	93	-	-
Ozdemir et al. [265]	ResNet50	C1	95.57	95.71	-	-
Ahuja et al. [266]	ResNet18	C9	98.07	95.66	98.83	-
Messaoud et al. [267]	VGG19	C1	86	79	-	-
Manconi et al. [268]	InceptionV1	C19	98.21	97.17	99.24	99.72
Cheng et al. [269]	VBNet + LSTM	Private	89	84	-	-
Lu et al. [270]	ResNet18	C15	97.78	97.94	97.65	-
Owais et al. [271]	DSSNet	C3	96.58	-	-	98.54
Yoo et al. [272]	2D UNet	Private	-	-	-	-
Suri et al. [273]	ResNet-UNet	Private	98	-	-	87.00
Ghose et al. [274]	DenseNet169TL	C22	99.95	-	99.97	-
Gunraj et al. [275]	CNNs	C25	99	99.1	-	-
Yousefzadeh et al. [276]	UNet + KNN	C9	-	-	-	-
Choudhary et al. [277]	ResNet34	C4	95.47	92.16	99.42	-
Chouat et al. [278] *	VGGNet19, Xception	C11	90.5, 89.5	-	-	-
Dialameh et al. [279]	DenseNet121	C3	-	85.8	-	-
Venkatachalam et al. [280]	CNN	Private	98.5	97	100	-
Latif et al. [281]	ResNet18 + GoogleNet2000 features with SVM	C19	99.9	-	-	-
El-Shafai et al. [282]	DCNN	C1	98.49	-	-	-
Xue et al. [283]	CNN	C1	97.67	-	-	-
El-Shafai et al. [284]	CNN	C9	100	-	-	-

* Other data collections were also used.

**Table 5:** Comparison: COVID-19 detection using CNN architecture according to the year of publication.

Author [Ref] (Year)	Dataset Collection	Performance (%)			
		ACC	SPEC	SEN	AUC
Song et al. [63] (2020)	Private	-	91	92	97
Singh et al. [65] (2020)	Private	93.5	~90	~90	-
Ning et al. [66] (2020)	C17	-	-	-	89.6
Babukarthik et al. [68] (2020) *	C23	98.84	97.0	100	-
Han et al. [70] (2020)	Private	97.9	-	-	99
Gunraj et al. [72] (2020)	C3	99.1	99.9	97.3	-
Heidarian et al. [140] (2020)	C22	90.82	86.04	94.55	98.0
Mishra et al. [73] (2020)	C1	86	-	-	88.3
Qian et al. [79] (2020)	Private	-	97.49	98.99	99.93
Li et al. [80] (2020)	Private	-	-	-	96.0
Silva et al. [75] (2020)	C1, C4	87.68	-	-	-
Jin et al. [82] (2020) *	C9, C20	94.98	95.76	90.19	97.71
Jamshidi et al. [83] (2020) *	C1, C16, C25	98.49	-	-	-
Zhang et al. [85] (2020)	Private	-	-	-	95.9
Owais et al. [195] (2020) *	C2, C9	97.60	99.29	90.32	98.65
Misztal et al. [89] (2020)	C1, C19	-	-	-	-
Polsinelli et al. [91] (2020)	C6	85.03	81.95	87.55	-
Masud et al. [212] (2020)	C16	96	-	-	99
Hu et al. [96] (2020)	Private	87.1	91.23	80.83	90.6
Kang et al. [99] (2020)	private	95.5	96.6	93.2	-
Bai et al. [101] (2020)	Private	96	93.2	95	95
Zhu et al. [102] (2020)	C25	-	-	-	-
Zhang et al. [107] (2021)	Private	93.64 ± 1.42	94.00 ± 1.56	93.28 ± 1.50	-
Yao et al. [109] (2021)	Private	-	90.5	91.5	95.5
Acar et al. [110] (2021)	C2, C1	-	-	-	-
Ravi et al. [111] (2021) *	C16	99	-	-	-
Huang et al. [113] (2021) *	Private	98.28	-	-	-
Kundu et al. [117] (2021)	C4	97.81	97.77	97.81	-
Saad et al. [118] (2021)	C16	98.9	-	-	-
Lee et al. [127] (2021)	C5	-	-	-	80
Mishra et al. [128] (2021) *	C1	99	-	-	98.6
Barbosa et al. [130] (2021) *	Private	-	-	-	
Jadhav et al. [132] (2021)	Private	95.2	94.9	95.3	98.5
Yao et al. [134] (2021)	C1, C5	98.49	97.95	99.0	-
Khalifa et al. [138] (2021)	C8, C14	99.3	95	98.12	-
Bhuyan et al. [139] (2021) *	C2	99	99.41	96.66	-
Chaddad et al. [143] (2021) *	Private	82.80			88.16
Yousefzadeh et al. [144] (2021) *	C20, C9	-	92.7	94.5	95.6
Jiang et al. [148] (2021)	C1	96	-	-	98.89
Pathan et al. [154] (2021)	C1	96	96	97	-
Cruz et al. [155] (2021)	C1	86.70	-	89.52	90.82
Basset et al. [157] (2021) *	C22	96.80	-	-	98.86
Aslan et al. [159] (2021) *	C19	98.70	-	-	99
Kundu et al. [160] (2021)	C3	98.93	98.93	98.93	-
Zhang et al. [163] (2021)	Private	98.02	97.95	98.10	-
Chen et al. [166] (2021)	C10	96.7	-	95.6	-
Alshazly et al. [167] (2021)	C1, C4	96.15	96.75	95.9	-
Rajasekar et al. [170] (2021)	C3	94.89	95	-	-
Xie et al. [171] (2021) *	Private	-	80.0	83.6	90.6
Garain et al. [174] (2021)	C1	99.51	100	98.96	-
Elghamrawy et al. [175] (2021)	Private	98	-	98.8	96
Sen et al. [176] (2021)	C4, C1	94.19	-	-	95.5
Teodoro et al. [177] (2021)	C4	-	98.53	98.53	-
Yasar et al. [178] (2021)	C1, C4, C3	95.16	94.01	97.54	99.06
Afshar et al. [180] (2021)	Private	93	-	--	-
Kundu et al. [182] (2021)	C4, C26	98.86	98.86	98.87	-
Pal et al. [183] (2021) *	C1	84	-	-	-
Helwan et al. [185] (2021)	Private	98.7	97.3	98.1	-
Castiglione et al. [186] (2021)	C4	99.99	99.97	99.92	-
Yan et al. [187] (2021)	Private	-	-	75.1	-
Wan et al. [190] (2021)	Private	94.75	96.69	93.22	-
Polat et al. [193] (2021) *	C6	93.26	93.24	92.37	-
Naeem et al. [199] (2021) *	C4, C6	90.98	-	-	-
Fouladi et al. [201] (2021)	C4	93.3	-	87.67	-
Wang et al. [202] (2021)	Private	96.66	-	-	-
Siddiqui et al. [207] (2021)	Private	95.54	97.06	94.38	-
Haikel et al. [208] (2021)	C4, C1	99.72	-	99.80	99.99
Bekhet et al. [209] (2021) *	Private	92.08	-	-	-
El-Shafai et al. [211] (2021)	C1, C25, C16	99.05	-	-	-
El-Shafai et al. [213] (2021)	C16	100	-	-	-
Liu et al. [230] (2022)	C1, C4	97.21 and 97.94	95.76 and 95.43	96.21 and 95.22	-
Saheb et al. [232] (2022)	C3	98.49	96.83	96.83	-
Bhuyan et al. [139] * (2022)	C1	99	99.26	95.82	-
Shaik et al. [240] (2022)	C1, C4	93.33	-	93.25	93.25
Reis et al. [241] (2022)	C4	97.60	-	100	-
Fan et al. [243] (2022)	C25	96.73	96.01	97.76	-
Karthik et al. [244] (2022)	C9	-	-	-	-
Smadi et al. [246] (2022)	C4	98.79	98.8	98.8	-
Li et al. [275] (2022)	Private	92.647	-	93.323	-
Yang et al. [255] (2022)	C4	97.55	98.14	96.41	-
Mijares et al. [256] (2022)	C4	94.89	-	90.43	-
Heidari et al. [257] (2022)	Private	99.76	-	99.40	-
Joshi et al. [261] (2022)	C1, and C4	96.13	-	-	96.13
Gunraj et al. [275] (2022)	C25	99	-	99.1	-
Venkatachalam et al. [280] (2022)	Private	98.5	100	97	-
Xue et al. [283] (2022)	C1	97.67	-	-	-
El-Shafai et al. [284] (2022)	C9	100	-	-	-

* Other data collections were also used.

**Table 6:** Comparison: COVID-19 detection using ResNet architecture according to the year of publication.

Author [Ref] (Year)	Dataset Collection	Performance (%)			
		ACC	SPEC	SEN	AUC
Song et al. [108] (2020)	Private	86	77	-	95
Loey et al. [62] (2020)	C1	82.91	87.62	77.66	-
Mohammed et al. [69] (2020)	C19	77.6	79.3	85.5	-
Chen et al. [100] (2020)	C1, C6, C2	86.8	-	-	93.1
Javor et al. [74] (2020)	Private	-	93.3	84.4	95.6
Pathak et al. [76] (2020) *	C1	93.0	91.4	94.7	-
Ko et al. [92] (2020) *	C6	96.97	100	99.58	-
Wang et al. [94] (2020)	93.3	-	95.5	97.3	-
Jin et al. [82] (2020) *	C9, C20	94.98	95.76	90.19	97.71
Sharma et al. [104] (2020) *	C1, C9	91	-	-	-
Li et al. [80] (2020)	Private	-	96	90	96
Wang et al. [98] (2020)	Private	85.00	79.35	71.43	90.11
Goncharov et al. [106] (2021) *	C2	-	-	-	0.97 ± 0.01
Chen et al. [112] (2021) *	Private	91.21	88.46	94.87	-
Lascu et al. [121] (2021) *	C1	94.9	-	-	-
Yan et al. [124] (2021)	C4	96.3	-	-	-
Shalbaf et al. [125] (2021)	C1	85	-	85	-
Rahimzadeh et al. [126] (2021)	C3	98.49	-	94.96	-
Zhu et al. [136] (2021) *	C1	93	92	93	-
Wang et al. [147] (2021) *	Private	-	-	96.25	-
Jingxin et al. [150] (2021) *	C1, C19	97.83	-	96.89	-
Ahamed et al. [153] (2021) *	C15	99.99	-	-	-
Mondal et al. [165] (2021)	C2, C4	96.18	97.96	-	-
Biswas et al. [184] (2021)	C4	98.79	-	-	98.80
Suri et al. [188] (2021)	Private	-	-	-	96.75
Nair et al. [189] (2021)	C4	-	96	90	95
Guo et al. [191] (2021)	Private	99.34	-	-	-
Jia et al. [196] (2021) *	C25	99.3	-	-	-
Murugan et al. [198] (2021)	C4	98.78	99.19	98.37	-
Yu et al. [203] (2021)	Private	96.62	95.91	97.33	-
Wang et al. [215] (2021)	Private	-	99.2	97.4	99.1
Ahuja et al. [216] (2021) *	C1	99.4	98.6	100	99.65
Benbrahim et al. [103] (2021)	Private	99.01	100	72	-
Khurana and Soni* [221] (2022)	C23	98.9	99.2	98.6	-
Canayaz et al. [222] (2022)	C1 and C4	95.79	95.75	95.83	-
Zouch et al. [224] (2022) *	C1	98.06	-	-	-
Habib et al. [226] * (2022)	Not provided	99.30	-	99.10	-
Cao et al. [234] (2022)	Private	82.7	-	79.1	-
Jingxin et al. [150] (2022)	C8, C18, C1	98.39	-	-	-
Fallahpoor et al. [247] (2022)	Private	85.00	-	-	-
Q et al. [252] * (2022)	C2	-	-	-	-
Qi et al. [253] (2022)	C19	93.4	-	-	87.6
Oğuz and Yağanoğlu [254] (2022)	Private	96.29	-	95.082	98.21
Sangeetha et al. [259] (2022)	C15	98	-	-	-
Mohammed et al. [260] (2022)	C1	91.46	-	-	-
Dara et al. [264] (2022)	C1, C2, C9	97.53	-	93	-
Ozdemir et al. [265] (2022)	C1	95.57	-	95.71	-
Ahuja et al. [266] (2022)	C9	98.07	98.83	95.66	-
Lu et al. [270] (2022)	C15	97.78	97.65	97.94	-
Suri et al. [273] (2022)	Private	98	-	-	87.00
Choudhary et al. [277] (2022)	C4	95.47	99.42	92.16	
Latif et al. [281] (2022)	C19	99.9	-	-	-

* Other data collections were also used.

**Table 7:** Comparison: COVID-19 detection using VGG architecture according to the year of publication.

Authors [Ref] (Year)	Data Collection	Performance (%)			
		ACC	SPEC	SEN	AUC
Panwar et al. [88] (2020) *	C4, C25	95.61	97.22	76	-
Deng et al. [95] (2020)	C25	75	-	-	-
Zhu et al. [102] (2020)	C25	-	-	-	-
Ibrahim et al. [105] (2021) *	C6	98.05	99.5	-	99.66
Jangam et al. [114] (2021) *	C1, C3, C4	91.49	-	-	-
Tan et al. [120] (2021)	C1	98	94.9	99	-
Guiot et al. [133] (2021) *	Private	85.18	91.63	69.52	88.2
Singh et al. [135] (2021)	C20	99.29	-	-	-
Hu et al. [149] (2021)	C1, C3, C2	95.43	-	-	-
Balaha et al. [151] (2021)	C1, C3, C24	99.33	-	-	-
Khan et al. [169] (2021)	C8	98	-	-	99
Sethy et al. [172] (2021) *	Private	64.80	-	-	-
Brahim et al. [179] (2021)	C9	97.67	98.41	97.57	-
Biswas et al. [184] (2021)	C4	98.79	-	-	98.80
Suri et al. [188] (2021)	Private	-	-	-	96.75
Li et al. [194] (2021)	C20	93.57	94.21	93.93	-
Lacerda et al. [206] * (2021)	C9	88	-	97	-
Kaushik et al. [210] (2021)	C4	95.26	95.10	95.30	-
Subhalakshmi et al. [223] (2022)	C1	96.81	95.81	96.53	-
Zouch et al. [224] (2022) *	C1	98.06	-	-	-
Pandey et al. [229] (2022)	C15	99.28	-	-	-
Ibrahim et al. [236] (2022)	C1	95	96	95	-
Florescu et al. [238] (2022)	Private	1.57	-	-	-
Sangeetha et al. [259] (2022)	C15	98	-	-	-
Zhang et al. [262] (2022)	Private	94.12	96.95	91.40	97.44
Messaoud et al. [267] (2022)	C1	86	-	79	-
Chouat et al. [278] * (2022)	C11	90.5	-	-	-

* Other data collections were also used.

**Table 8:** Comparison: COVID-19 detection using DenseNet architecture according to the year of publication.

Author [Ref] (Year)	Dataset Collection	Performance (%)			
		ACC	SPEC	SEN	AUC
Hu et al. [61] (2020)	C1	86.00	-	-	94.00
Jaiswal et al. [67] (2020)	C4	96.25	96	96	-
Peng et al. [78] (2020) *	C13	-	-	78.0	89.1
Jin et al. [82] (2020)	Private	90.8	93	84	-
Liu et al. [87] (2020)	C1, C17	-	-	-	76.09
Jangam et al. [114] (2021) *	C1, C3, C4	91.49	-	-	-
Wang et al. [147] (2021) *	Private	-	-	96.25	-
Li et al. [162] (2021)	C1	87	-	-	75
Liu et al. [181] (2021) *	Private	94.3	88.50	91.12	98
Kassania et al. [214] (2021) *	C25	99.0	99.0	99.0	-
Habib et al. [226] * (2022)	Not provided	99.3	-	99.1	-
Sadik et al. [248] (2022)	C19	93.8	90.0	97.5	-
Ghose et al. [274] (2022)	C22	99.95	99.97	-	
Dialameh et al. [279] (2022)	C3	-	-	85.8	-

* Other data collections were also used.

**Table 9:** Comparison: COVID-19 detection using Inception architecture according to the year of publication.

Authors [Ref] (Year)	Dataset Collection	Performance (%)			
		ACC	SPEC	SEN	AUC
El-Bana et al. [93] (2020) *	Private	99.5	99.2	99.8	-
Benbrahim et al. [103] (2020)	Private	99.01	100	72	-
Shalbaf et al. [125] (2021)	C1	85	-	85	-
Wang et al. [220] (2021)	Private	79.30	83	67	-
Subhalakshmi et al. [223] (2022)	C1	96.81	95.81	96.53	-
Montalbo et al. [227] * (2022)	C22, C2, C9, C1, C18	97.41	-	97.52	99.0
Batra et al. [233] (2022)	C1	93	-	89.81	-
Ortiz et al. [258] * (2022)	C19	91	-	33	80.0
Manconi et al. [268] (2022)	C19	98.21	99.24	97.17	99.72

* Other data collections were also used.

**Table 10:** Comparison: COVID-19 detection using UNet architecture according to the year of publication.

Authors [Ref] (year)	Dataset Collection	Performance (%)			
		ACC	SPEC	SEN	AUC
Ni et al. [60] (2019)	Private	Per lobe—83 Per patient—94	-	96 100	86.54 86.08
Chaganti et al. [64] (2020)	Private	-	-	-	-
Alirr et al. [116] (2021)	C2, C6	-	95.1	82.2	-
Fung et al. [119] (2021)	C2	-	-	-	98.66
Zhang et al. [129] (2021)	Private	-	-	-	-
Jiang et al. [71] (2020)	C2	-	-	-	-
Kuchana et al. [137] (2021)	C14, C2	-	-	-	-
Munusamy et al. [146] (2021)	C2	99	-	-	-
Hasan et al. [156] (2021) *	C4, C1	90.14	88.59	-	94.60
Basset et al. [157] (2021) *	C22	96.80	-	-	98.86
Müller et al. [161] (2021) *	C25	-	-	-	-
Xu et al. [164] (2021) *	C2	96.1	-	78.6	-
Lessmann et al. [81] * (2020)	Private	-	89.8	85.7	95
Voulodimos et al. [168] (2021) *	C2, C8	-	-	-	-
Wang et al. [84] (2020) *	Private	90.1	-	-	95.9
Amyar et al. [90] (2020)	C1, C2	94.67	92	96	-
He et al. [197] (2021)	Private	98.5	-	-	99.1
Kalane et al. [200] (2021)	C1, C6	94.10	93.47	94.86	-
Gao et al. [204] (2021)	Private	94.80	94.45	95.42	98.17
Wang et al. [215] (2021)	Private	-	99.2	97.4	99.1
Kang et al. [99] (2020)	Private	95.5	96.6	93.2	-
Chen et al. [100] (2020)	Private	98.85	99.16	94.34	-
Pu et al. [217] (2021)	Private	-	84	95	-
Luo et al. [231] (2022)	Private	93.84	-	93.15	-
Suri et al. [273] (2022)	Private	98.00	-	-	87.00
Yoo et al. [272] (2022)	Private	-	-	-	-
Yousefzadeh et al. [276] (2022)	C9		-	-	-

* Other data collections were also used.

**Table 11:** Comparison: COVID-19 detection using MobileNet architecture according to the year of publication.

Authors [Ref] (Year)	Dataset Collection	Performance (%)			
		ACC	SPEC	SEN	AUC
Singh et al. [135] (2021)	C4	96.40	-	98	99.5
Canayaz et al. [222] (2022)	C1 and C4	99.06	95.75	95.83	-
Singh and Kolekar [115] (2022)	C4	96	-	98	-

**Table 12:** Comparison: COVID-19 detection using EfficientNet architecture according to the year of publication.

Authors [Ref] (Year)	Dataset Collection	Performance (%)			
		ACC	SPEC	SEN	AUC
Balaha et al. [225] (2022)	C18	99.61	-	99.62	99.98
Garg et al. [242] (2022)	C4	98.45	98.83	96.82	-
Verma et al. [245] (2022)		99.58	-	99.69	-
Huang et al. [249] (2022)	C1, C4	97.48	-	-	-
Ravi et al. [111] (2022)	C16	99.00	-	99.00	-

**Table 13:** Data augmentation techniques according to year of publication.

Author [Ref] (Year)	Used Methods	Motivation	Dataset Collection	Dataset (Augmentation)	Performance (%)			
					ACC	SEN	SPEC	AUC
Loey et al. [62] (2020)	CGAN	Model accuracy improvement	C1	1502 (4843)	82.91	77.66	87.62	-
Mohammed et al. [69] (2020)	Contrast stretching, the addition of Gaussian noise, blur, and spatial transformations such as zooming, scaling, rotation, and elastic deformation	Augment positive samples count	C19	22,873 (-)	77.6	85.5	79.3	-
Jiang et al. [71] (2020)	GAN (random resizing and cropping, random rotation, Gaussian noise, and elastic transform)	Increase image number	C2	373 (2220)	-	-	-	-
Chen et al. [145] (2020)	Random cropping, resizing, color distortion	Classification performance improvement	C1, C2	1286 (-)	86.8	-	-	93.1
Silva et al. [75] (2020)	Rotation (0–15° clockwise or anticlockwise), horizontal flip, scaling, 20% zoom	Increase data size	C1, C4	1693 (-)	87.68	-	-	-
Wang et al. [84] (2020)	Random affine transformation (horizontal and vertical translations, shearing in the width dimension) and color jittering (adjusted brightness and contrast)	Avoid overfitting	Private	- (310,055)	90.1	-	-	95.9
Zhang et al. [85] (2020)	Crop square patches, rotation with an angle, random horizontal reflection, and adjusted contrast by random darkening and brightening	Increase number of images	Private	- (630)	-	-	-	95.9
Ko et al. [92] (2020)	Rotation between –10° and 10° and 90% (zoom-in) and 110% (zoom-out)	Increase number of images	Private	- (3993)	96.97	99.58	100	-
El-Bana et al. [93] (2020)	Crop square patches, rotation with an angle (Δ = −25 to 25), random horizontal reflection, adjust contrast (factor ranging from 0.5 to 1.5)	Avoid overfitting	Private	- (499)	99.5	99.8	99.2	-
Hu et al. [96] (2020)	Cropping square patches, rotation with an angle of −25 to + 25 degrees, random horizontal reflection, and contrast adjustment (factor ranging between 0.5 and 1.5)	Increase dataset size	Private	- (-)	87.1	80.83	91.23	90.6
Ibrahim et al. [105] (2021)	Resizing, rotating, flipping, skewing	Increase number of Images	C6	33,676 (75,000)	98.05	-	99.5	99.66
Acar et al. [110] (2021)	GAN	Increase effectiveness of DL models	C2, C1	1607 (3921)	-	-	-	-
Huang et al. [113] (2021)	Vertical–horizontal flip, rotation (90, 180, 270 degrees)	Acquire richer samples	FaNet	422 (12,924)	98.28	-	-	-
Jangam et al. [114] (2021)	Random resized crop, rotation, horizontal flip, color jittering	Increase size of dataset	C1, C3, C4	15,286 (-)	91.49	-	-	-
Tan et al. [120] (2021)	SRGAN	Enhancement in model accuracy	C1	746(-)	97.9	99	94.9	-
Lascu et al. [121] (2021)	Random patching, resized	Generate more samples	C1	746 (-)	94.9	-	-	-
Bhuyan et al. [139] (2021)	RAIOSS	Changing image	C2	- (3855)	99	96.66	99.41	-
Wang et al. [147] (2021)	Noise injection, HS transform, vs. transform, rotation, GC, RT, and scaling	Improve generalization of model	Private	1164 (-)	-	96.25	-	-
Jiang et al. [148] (2021)	CycleGAN	Increase data size	C1	600 (2000)	96	-	-	98.89
Jingxin et al. [150] (2021)	Coronal view, squeezing	Improve model performance	C1, C19	-(-)	97.83	96.89	-	-
Balaha et al. [151] (2021)	Cropping, zooming, shearing, rotating, flipping, and changing the brightness	Increase Data size	C1, C3, C24	- (15,535)	99.33	-	-	-
Turkoglu et al. [152] (2021)	Symmetrical rotation (90 and 270 degree), reflection	Increase classification accuracy	C1	746 (3730)	98.36	98.28	98.44	98.36
Ahamed et al. [153] (2021) *	Rescaling, zooming, horizontal flipping and shearing operations	Reduce network generalization error	C15	3000 (7593)	99.99	-	-	-
Aslan et al. [159] (2021)	Crop, rotation	Increase number of images	C19	- (1095)	98.70	-	-	99
Zhang et al. [163] (2021)	Salt and pepper noise (SAPN) and speckle noise	Avoid overfitting	Private	- (-)	98.02	98.10	97.95	-
El-Shafai et al. [211] (2021)	GAN	Improve model accuracy	C1, C25, C16	- (-)	99.05	-	-	-
Zouch et al. [224] (2022)	Random rotation with angle ranging from +20 to -20 degrees, random noise, horizontal flip	Increase dataset size	C1	- (-)	76.32 (ResNet50) and 84.87 (VGG19)	-	-	-
Balaha et al. [225] (2022)	GANs, CycleGAN, CCGAN	Avoid overfitting	C18	- (-)	99.57 and 99.14	-	-	-
Habib et al. [226] (2022)	Contrast enhancement	developed robust system	Not mentioned	21,165 (47,440)	99.3	99.1	-	
Bhuyan et al. [139] (2022)	RAIOSS	generated different quality images	C1	746 (3855)	99	95.82	99.26	
Ibrahim et al. [236] (2022)	Rotation of all images to 90, 180, 270	To increase number of images and attain high generalizability	C1	746 (2984)	95	95	96	
Akinyelu et al. [237] (2022)	Rotation, zoom, width shift, height shift, shear	Increase dataset size	Private	- (194,922)	97.50 (B), 99.86	100, 99.83	93.19, 99.90	
Baghdadi et al. [239] * (2022)	Rotation, width shift ratio, height shift ratio, shear ratio, zoom ratio, brightness change, vertical flip, horizontal flip	To balance datasets	C15	14,486 (15,186)	99.73	-	-	
Karthik et al. [244] (2022)	Random rotation, random translation, shearing, horizontal flip	Improve generalizability	C9	-	-	-	-	
Fallahpoor et al. [247] (2022)	Random rotation, 90 degree rotation, scaling, translation	Prevent overfitting and improve model performance	Private	-	-	-	-	
Joshi et al. [261] (2022)	Horizontal flip, anticlockwise rotation (5 degree angle), clockwise rotation (5 degree angle), gaussian noise	Increase sample size	C1, and C4	-	93.59, 98.79	-	-	
Chouat et al. [278] (2022)	Scaling, rotating, shifts, and flips	Increase data and improve network efficiency	C11	-	90.5, 89.5	-	-	
El-Shafai et al. [284] (2022)	Rotation, width shift range, feature wise center, sample-wise center, brightness adjustment	Increase sample size	C1	-	98.49	-	-	

## Data Availability

Not applicable.

## References

[1] Wu F., Zhao S., Yu B., Chen Y.-M., Wang W., Song Z.-G., Hu Y., Tao Z.-W., Tian J.-H., Pei Y.-Y. (2020). A new coronavirus associated with human respiratory disease in China. Nature.

[2] Cucinotta D., Vanelli M. (2020). Who declares COVID-19 a pandemic. Acta Biomed..

[3] Worldometers. https://www.worldometers.info/coronavirus/.

[4] Santosh K., Ghosh S., GhoshRoy D. (2022). Deep Learning for COVID-19 Screening Using Chest X-rays in 2020: A Systematic Review. Int. J. Pattern Recognit. Artif. Intell..

[5] Nishiura H., Linton N.M., Akhmetzhanov A.R. (2020). Serial interval of novel coronavirus (COVID-19) infections. Int. J. Infect. Dis..

[6] Santosh K.C. (2020). COVID-19 Prediction Models and Unexploited Data. J. Med. Syst..

[7] Huang C., Wang Y., Li X., Ren L., Zhao J., Hu Y., Zhang L., Fan G., Xu J., Gu X. (2020). Clinical features of patients infected with 2019 novel coronavirus in Wuhan, China. Lancet.

[8] Razai M.S., Doerholt K., Ladhani S., Oakeshott P. (2020). Coronavirus disease 2019 (COVID-19): A guide for UK GPs. BMJ.

[9] Remuzzi A., Remuzzi G. (2020). COVID-19 and Italy: What next?. Lancet.

[10] Sohrabi C., Alsafi Z., O’Neill N., Khan M., Kerwan A., Al-Jabir A., Iosifidis C., Agha R. (2020). World Health Organization declares global emergency: A review of the 2019 novel coronavirus (COVID-19). Int. J. Surg..

[11] Mahbub M.K., Biswas M., Gaur L., Alenezi F., Santosh K. (2022). Deep features to detect pulmonary abnormalities in chest x-rays due to infectious diseasex: COVID-19, pneumonia, and tuberculosis. Inf. Sci..

[12] Santosh K., Gaur L. (2021). Artificial Intelligence and Machine Learning in Public Healthcare Opportunities and Societal Impact.

[13] Santosh K., Das N., Ghosh S. (2021). Deep Learning Models for Medical Imaging.

[14] Islam M., Rahaman A., Islam R. (2020). Development of Smart Healthcare Monitoring System in IoT Environment. SN Comput. Sci..

[15] Rahaman A., Islam M.M., Islam M.R., Nooruddin S. (2019). Developing IoT Based Smart Health Monitoring Systems: A Review. Rev. D’intelligence Artif..

[16] Kumar A., Gupta P.K., Srivastava A. (2020). A review of modern technologies for tackling COVID-19 pandemic. Diabetes Metab. Syndr. Clin. Res. Rev..

[17] Ting D.S.W., Carin L., Dzau V., Wong T.Y. (2020). Digital technology and COVID-19. Nat. Med..

[18] Rubin G.D., Ryerson C.J., Haramati L.B., Sverzellati N., Kanne J.P., Raoof S., Schluger N.W., Volpi A., Yim J.-J., Martin I.B.K. (2020). The Role of Chest Imaging in Patient Management during the COVID-19 Pandemic: A Multinational Consensus Statement from the Fleischner Society. Radiology.

[19] Dong D., Tang Z., Wang S., Hui H., Gong L., Lu Y., Xue Z., Liao H., Chen F., Yang F. (2020). The Role of Imaging in the Detection and Management of COVID-19: A Review. IEEE Rev. Biomed. Eng..

[20] Zhao D., Yao F., Wang L., Zheng L., Gao Y., Ye J., Guo F., Zhao H., Gao R. (2022). A comparative study on the clinical features of COVID-19 pneumonia to other pneumonias. Clinical Infectious Diseases.

[21] Schalk A.F., Hawn M.C. (1931). An apparent new respiratory disease of baby chicks. J. Am. Vet. Med. Assoc..

[22] Fabricant J. (1998). The Early History of Infectious Bronchitis. Avian Dis..

[23] Cook J.K.A., Jackwood M., Jones R.C. (2012). The long view: 40 years of infectious bronchitis research. Avian Pathol..

[24] Drosten C., Günther S., Preiser W., Van Der Werf S., Brodt H.-R., Becker S., Rabenau H., Panning M., Kolesnikova L., Fouchier R.A.M. (2003). Identification of a Novel Coronavirus in Patients with Severe Acute Respiratory Syndrome. N. Engl. J. Med..

[25] Corman V.M., Muth D., Niemeyer D., Drosten C. (2018). Hosts and Sources of Endemic Human Coronaviruses. Adv. Virus Res..

[26] Zaki A.M., Van Boheemen S., Bestebroer T.M., Osterhaus A.D.M.E., Fouchier R.A.M. (2012). Isolation of a Novel Coronavirus from a Man with Pneumonia in Saudi Arabia. N. Engl. J. Med..

[27] McIntosh K., Dees J.H., Becker W.B., Kapikian A.Z., Chanock R.M. (1967). Recovery in tracheal organ cultures of novel viruses from patients with respiratory disease. Proc. Natl. Acad. Sci. USA.

[28] Mackay I.M., Arden K.E. (2015). MERS coronavirus: Diagnostics, epidemiology and transmission. Virol. J..

[29] Boopathi S., Poma A.B., Kolandaivel P. (2020). Novel 2019 coronavirus structure, mechanism of action, antiviral drug promises and rule out against its treatment. J. Biomol. Struct. Dyn..

[30] Khan S., Fakhar Z., Hussain A., Ahmad A., Jairajpuri D.S., Alajmi M.F., Hassan M.I. (2020). Structure-based identification of potential SARS-CoV-2 main protease inhibitors. J. Biomol. Struct. Dyn..

[31] Shi F., Wang J., Shi J., Wu Z., Wang Q., Tang Z., He K., Shi Y., Shen D. (2020). Review of artificial intelligence techniques in imaging data acquisition, segmentation and diagnosis for COVID-19. IEEE Rev. Biomed. Eng..

[32] McCall B. (2020). COVID-19 and artificial intelligence: Protecting health-care workers and curbing the spread. Lancet Digit. Health.

[33] Vaishya R., Javaid M., Khan I.H., Haleem A. (2020). Artificial Intelligence (AI) applications for COVID-19 pandemic. Diabetes Metab. Syndr. Clin. Res. Rev..

[34] Liberati A., Altman D.G., Tetzlaff J., Mulrow C., Gøtzsche P.C., Ioannidis J.P.A., Clarke M., Devereaux P.J., Kleijnen J., Moher D. (2009). The PRISMA statement for reporting systematic reviews and meta-analyses of studies that evaluate health care interventions: Explanation and elaboration. PLoS Med..

[35] Chen H., Engkvist O., Wang Y., Olivecrona M., Blaschke T. (2018). The rise of deep learning in drug discovery. Drug Discov. Today.

[36] Esteva A., Robicquet A., Ramsundar B., Kuleshov V., DePristo M., Chou K., Cui C., Corrado G., Thrun S., Dean J. (2019). A guide to deep learning in healthcare. Nat. Med..

[37] Wainberg M., Merico D., Delong A., Frey B.J. (2018). Deep learning in biomedicine. Nat. Biotechnol..

[38] Shen D., Wu G., Suk H.-I. (2017). Deep learning in medical image analysis. Annu. Rev. Biomed. Eng..

[39] LeCun Y., Bengio Y., Hinton G. (2015). Deep learning. Nature.

[40] Goodfellow I., Bengio Y., Courville A., Bengio Y. (2016). Deep Learning.

[41] Tang X. (2019). The role of artificial intelligence in medical imaging research. BJR|Open.

[42] Ranschaert E.R., Morozov S., Algra P.R. (2019). Artificial Intelligence in Medical Imaging: Opportunities, Applications and Risks.

[43] Desai S.B., Pareek A., Lungren M.P. (2020). Deep learning and its role in COVID-19 medical imaging. Intell. Med..

[44] Gaur U., Majumder A.A., Sa B., Sarkar S., Williams A., Singh K. (2020). Challenges and Opportunities of Preclinical Medical Education: COVID-19 Crisis and Beyond. SN Compr. Clin. Med..

[45] Tayarani M. (2020). Applications of artificial intelligence in battling against COVID-19: A literature review. Chaos Solitons Fractals.

[46] Islam M., Karray F., Alhajj R., Zeng J. (2021). A Review on Deep Learning Techniques for the Diagnosis of Novel Coronavirus (COVID-19). IEEE Access.

[47] Fan D.-P., Zhou T., Ji G.-P., Zhou Y., Chen G., Fu H., Shen J., Shao L. (2020). Inf-Net: Automatic COVID-19 Lung Infection Segmentation From CT Images. IEEE Trans. Med. Imaging.

[48] Shorten C., Khoshgoftaar T.M., Furht B. (2021). Deep Learning applications for COVID-19. J. Big Data.

[49] Ouyang X., Huo J., Xia L., Shan F., Liu J., Mo Z., Yan F., Ding Z., Yang Q., Song B. (2020). Dual-Sampling Attention Network for Diagnosis of COVID-19 From Community Acquired Pneumonia. IEEE Trans. Med. Imaging.

[50] Zheng T., Oda M., Wang C., Moriya T., Hayashi Y., Otake Y., Hashimoto M., Akashi T., Mori M., Takabatake H. (2021). Unsupervised segmentation of covid-19 infected lung clinical ct volumes using image inpainting and representation learning. Medical Imaging 2021: Image Processing.

[51] Brooks N.A., Puri A., Garg S., Nag S., Corbo J., El Turabi A., Kaka N., Zemmel R.W., Hegarty P.K., Kamat A.M. (2021). The association of coronavirus disease-19 mortality and prior bacilli calmette-guerin vaccination: A robust ecological analysis using unsupervised machine learning. Sci. Rep..

[52] Sallay H., Bourouis S., Bouguila N. (2020). Online Learning of Finite and Infinite Gamma Mixture Models for COVID-19 Detection in Medical Images. Computers.

[53] Huang G., Liu Z., Van Der Maaten L., Weinberger K.Q. Densely connected convolutional networks. Proceedings of the IEEE Conference on Computer Vision and Pattern Recognition.

[54] Simonyan K., Zisserman A. (2014). Very deep convolutional networks for large-scale image recognition. arXiv.

[55] Szegedy C., Liu W., Jia Y., Sermanet P., Reed S., Anguelov D., Erhan D., Vanhoucke V., Rabinovich A. Going deeper with convolutions. Proceedings of the IEEE Conference on Computer Vision and Pattern Recognition.

[56] Howard A.G., Zhu M., Chen B., Kalenichenko D., Wang W., Weyand T., Andreetto M., Adam H. (2017). Mobilenets: Efficient convolutional neural networks for mobile vision applications. arXiv.

[57] He K., Zhang X., Ren S., Sun J. Deep residual learning for image recognition. Proceedings of the IEEE Conference on Computer Vision and Pattern Recognition.

[58] Boser B., Denker J.S., Henderson D., Howard R.E., Hubbard W., Jackel L.D., Laboratories H.Y.L.B., Zhu Z., Cheng J., Zhao Y. (1989). Backpropagation Applied to Handwritten Zip Code Recognition. Neural Comput..

[59] Ronneberger O., Fischer P., Brox T. (2015). U-net: Convolutional networks for biomedical image segmentation. International Conference on Medical Image Computing and Computer-Assisted Intervention.

[60] Ni Q., Sun Z.Y., Qi L., Chen W., Yang Y., Wang L., Zhang X., Yang L., Fang Y., Xing Z. (2020). A deep learning approach to characterize 2019 coronavirus disease (COVID-19) pneumonia in chest CT images. Eur. Radiol..

[61] He X. (2020). Sample-efficient deep learning for COVID-19 diagnosis based on CT scans. medRxiv.

[62] Loey M., Manogaran G., Khalifa N.E.M. (2020). A deep transfer learning model with classical data augmentation and CGAN to detect COVID-19 from chest CT radiography digital images. Neural Comput. Appl..

[63] Song J., Wang H., Liu Y., Wu W., Dai G., Wu Z., Zhu P., Zhang W., Yeom K.W., Deng K. (2020). End-to-end automatic differentiation of the coronavirus disease 2019 (COVID-19) from viral pneumonia based on chest CT. Eur. J. Nucl. Med..

[64] Chaganti S., Grenier P., Balachandran A., Chabin G., Cohen S., Flohr T., Georgescu B., Grbic S., Liu S., Mellot F. (2020). Automated Quantification of CT Patterns Associated with COVID-19 from Chest CT. Radiol. Artif. Intell..

[65] Singh D., Kumar V., Vaishali, Kaur M. (2020). Classification of COVID-19 patients from chest CT images using multi-objective differential evolution–based convolutional neural networks. Eur. J. Clin. Microbiol. Infect. Dis..

[66] Ning W., Lei S., Yang J., Cao Y., Jiang P., Yang Q., Zhang J., Wang X., Chen F., Geng Z. (2020). Open resource of clinical data from patients with pneumonia for the prediction of COVID-19 outcomes via deep learning. Nat. Biomed. Eng..

[67] Jaiswal A., Gianchandani N., Singh D., Kumar V., Kaur M. (2020). Classification of the COVID-19 infected patients using DenseNet201 based deep transfer learning. J. Biomol. Struct. Dyn..

[68] Babukarthik R.G., Adiga V.A.K., Sambasivam G., Chandramohan D., Amudhavel J. (2020). Prediction of COVID-19 Using Genetic Deep Learning Convolutional Neural Network (GDCNN). IEEE Access.

[69] Mohammed A., Wang C., Zhao M., Ullah M., Naseem R., Wang H., Pedersen M., Cheikh F.A. (2020). Weakly-Supervised Network for Detection of COVID-19 in Chest CT Scans. IEEE Access.

[70] Han Z., Wei B., Hong Y., Li T., Cong J., Zhu X., Wei H., Zhang W. (2020). Accurate Screening of COVID-19 Using Attention-Based Deep 3D Multiple Instance Learning. IEEE Trans. Med. Imaging.

[71] Jiang Y., Chen H., Loew M., Ko H. (2020). COVID-19 CT Image Synthesis ith a Conditional Generative Adversarial Network. IEEE J. Biomed. Health Inform..

[72] Gunraj H., Wang L., Wong A. (2020). COVIDNet-CT: A Tailored Deep Convolutional Neural Network Design for Detection of COVID-19 Cases From Chest CT Images. Front. Med..

[73] Mishra A.K., Das S.K., Roy P., Bandyopadhyay S. (2020). Identifying COVID19 from Chest CT Images: A Deep Convolutional Neural Networks Based Approach. J. Health Eng..

[74] Javor D., Kaplan H., Kaplan A., Puchner S., Krestan C., Baltzer P. (2020). Deep learning analysis provides accurate COVID-19 diagnosis on chest computed tomography. Eur. J. Radiol..

[75] Silva P., Luz E., Silva G., Moreira G., Silva R., Lucio D., Menotti D. (2020). COVID-19 detection in CT images with deep learning: A voting-based scheme and cross-datasets analysis. Inform. Med. Unlocked.

[76] Pathak Y., Shukla P., Tiwari A., Stalin S., Singh S. (2020). Deep Transfer Learning Based Classification Model for COVID-19 Disease. IRBM.

[77] Wu X., Hui H., Niu M., Li L., Wang L., He B., Yang X., Li L., Li H., Tian J. (2020). Deep learning-based multi-view fusion model for screening 2019 novel coronavirus pneumonia: A multicentre study. Eur. J. Radiol..

[78] Peng Y., Tang Y., Lee S., Zhu Y., Summers R.M., Lu Z. (2020). COVID-19-CT-CXR: A Freely Accessible and Weakly Labeled Chest X-ray and CT Image Collection on COVID-19 From Biomedical Literature. IEEE Trans. Big Data.

[79] Qian X., Fu H., Shi W., Chen T., Fu Y., Shan F., Xue X. (2020). M^3^Lung-Sys: A Deep Learning System for Multi-Class Lung Pneumonia Screening From CT Imaging. IEEE J. Biomed. Health Inform..

[80] Li L., Qin L., Xu Z., Yin Y., Wang X., Kong B., Bai J., Lu Y., Fang Z., Song Q. (2020). Using artificial intelligence to detect COVID-19 and community-acquired pneumonia based on pulmonary CT: Evaluation of the diagnostic accuracy. Radiology.

[81] Lessmann N., Sánchez C.I., Beenen L., Boulogne L.H., Brink M., Calli E., Charbonnier J.P., Dofferhoff T., van Everdingen W.M., Gerke P.K. (2020). Automated assessment of CO-RADS and chest CT severity scores in patients with suspected COVID-19 using artificial intelligence. Radiology.

[82] Jin C., Chen W., Cao Y., Xu Z., Tan Z., Zhang X., Deng L., Zheng C., Zhou J., Shi H. (2020). Development and evaluation of an artificial intelligence system for COVID-19 diagnosis. Nat. Commun..

[83] Jamshidi M.B., Lalbakhsh A., Talla J., Peroutka Z., Hadjilooei F., Lalbakhsh P., Jamshidi M., La Spada L., Mirmozafari M., Dehghani M. (2020). Artificial Intelligence and COVID-19: Deep Learning Approaches for Diagnosis and Treatment. IEEE Access.

[84] Wang X., Deng X., Fu Q., Zhou Q., Feng J., Ma H., Liu W., Zheng C. (2020). A Weakly-Supervised Framework for COVID-19 Classification and Lesion Localization From Chest CT. IEEE Trans. Med. Imaging.

[85] Zhang J., Chu Y., Zhao N. (2020). Supervised framework for COVID-19 classification and lesion localization from chest CT. Ethiop. J. Health Dev..

[86] Lai Y., Li G., Wu D., Lian W., Li C., Tian J., Ma X., Chen H., Xu W., Wei J. (2020). 2019 Novel Coronavirus-Infected Pneumonia on CT: A Feasibility Study of Few-Shot Learning for Computerized Diagnosis of Emergency Diseases. IEEE Access.

[87] Liu Q., Leung C.K., Hu P. (2020). A Two-Dimensional Sparse Matrix Profile DenseNet for COVID-19 Diagnosis Using Chest CT Images. IEEE Access.

[88] Panwar H., Gupta P., Siddiqui M.K., Morales-Menendez R., Bhardwaj P., Singh V. (2020). A deep learning and grad-CAM based color visualization approach for fast detection of COVID-19 cases using chest X-ray and CT-Scan images. Chaos Solitons Fractals.

[89] Misztal K., Pocha A., Durak-Kozica M., Wątor M., Kubica-Misztal A., Hartel M. (2020). The importance of standardisation—COVID-19 CT & Radiograph Image Data Stock for deep learning purpose. Comput. Biol. Med..

[90] Amyar A., Modzelewski R., Li H., Ruan S. (2020). Multi-task deep learning based CT imaging analysis for COVID-19 pneumonia: Classification and segmentation. Comput. Biol. Med..

[91] Polsinelli M., Cinque L., Placidi G. (2020). A light CNN for detecting COVID-19 from CT scans of the chest. Pattern Recognit. Lett..

[92] Ko H., Chung H., Kang W.S., Kim K.W., Shin Y., Kang S.J., Lee J.H., Kim Y.J., Kim N.Y., Jung H. (2020). COVID-19 Pneumonia Diagnosis Using a Simple 2D Deep Learning Framework with a Single Chest CT Image: Model Development and Validation. J. Med. Internet Res..

[93] El-Bana S., Al-Kabbany A., Sharkas M. (2020). A multi-task pipeline with specialized streams for classification and segmentation of infection manifestations in COVID-19 scans. PeerJ Comput. Sci..

[94] Wang J., Bao Y., Wen Y., Lu H., Luo H., Xiang Y., Li X., Liu C., Qian D. (2020). Prior-Attention Residual Learning for More Discriminative COVID-19 Screening in CT Images. IEEE Trans. Med. Imaging.

[95] Deng X., Shao H., Shi L., Wang X., Xie T. (2020). An Classification–Detection Approach of COVID-19 Based on Chest X-ray and CT by Using Keras Pre-Trained Deep Learning Models. Comput. Model. Eng. Sci..

[96] Hu S., Gao Y., Niu Z., Jiang Y., Li L., Xiao X., Wang M., Fang E.F., Menpes-Smith W., Xia J. (2020). Weakly Supervised Deep Learning for COVID-19 Infection Detection and Classification from CT Images. IEEE Access.

[97] Xu X., Jiang X., Ma C., Du P., Li X., Lv S., Yu L., Chen Y., Su J., Lang G. (2020). Deep Learning System to Screen novel Coronavirus Disease 2019 Pneumonia. Engineering.

[98] Wang S., Zha Y., Li W., Wu Q., Li X., Niu M., Wang M., Qiu X., Li H., Yu H. (2020). A fully automatic deep learning system for COVID-19 diagnostic and prognostic analysis. Eur. Respir. J..

[99] Kang H., Xia L., Yan F., Wan Z., Shi F., Yuan H., Jiang H., Wu D., Sui H., Zhang C. (2020). Diagnosis of Coronavirus Disease 2019 (COVID-19) With Structured Latent Multi-View Representation Learning. IEEE Trans. Med Imaging.

[100] Chen J., Wu L., Zhang J., Zhang L., Gong D., Zhao Y., Chen Q., Huang S., Yang M., Yang X. (2020). Deep learning-based model for detecting 2019 novel coronavirus pneumonia on high-resolution computed tomography. Sci. Rep..

[101] Bai H.X., Wang R., Xiong Z., Hsieh B., Chang K., Halsey K., Tran T.M.L., Choi J.W., Wang D.-C., Shi L.-B. (2020). Artificial Intelligence Augmentation of Radiologist Performance in Distinguishing COVID-19 from Pneumonia of Other Origin at Chest CT. Radiology.

[102] Zhu J., Shen B., Abbasi A., Hoshmand-Kochi M., Li H., Duong T.Q. (2020). Deep transfer learning artificial intelligence accurately stages COVID-19 lung disease severity on portable chest radiographs. PLoS ONE.

[103] Benbrahim H., Hachimi H., Amine A. (2020). Deep transfer learning with apache spark to detect COVID-19 in chest X-ray images. Rom. J. Inf. Sci. Technol..

[104] Sharma S. (2020). Drawing insights from COVID-19-infected patients using CT scan images and machine learning techniques: A study on 200 patients. Environ. Sci. Pollut. Res..

[105] Ibrahim D.M., Elshennawy N.M., Sarhan A.M. (2021). Deep-chest: Multi-classification deep learning model for diagnosing COVID-19, pneumonia, and lung cancer chest diseases. Comput. Biol. Med..

[106] Goncharov M., Pisov M., Shevtsov A., Shirokikh B., Kurmukov A., Blokhin I., Chernina V., Solovev A., Gombolevskiy V., Morozov S. (2021). CT-Based COVID-19 triage: Deep multitask learning improves joint identification and severity quantification. Med. Image Anal..

[107] Zhang Y.-D., Satapathy S.C., Liu S., Li G.-R. (2021). A five-layer deep convolutional neural network with stochastic pooling for chest CT-based COVID-19 diagnosis. Mach. Vis. Appl..

[108] Song Y., Zheng S., Li L., Zhang X., Zhang X., Huang Z., Chen J., Zhao H., Jie Y., Wang R. (2021). Deep learning Enables Accurate Diagnosis of Novel Coronavirus (COVID-19) with CT images. medRxiv.

[109] Yao J.-C., Wang T., Hou G.-H., Ou D., Li W., Zhu Q.-D., Chen W.-C., Yang C., Wang L.-P., Fan L.-Y. (2021). AI detection of mild COVID-19 pneumonia from chest CT scans. Eur. Radiol..

[110] Acar E., Şahin E., Yılmaz I. (2021). Improving effectiveness of different deep learning-based models for detecting COVID-19 from computed tomography (CT) images. Neural Comput. Appl..

[111] Ravi V., Narasimhan H., Chakraborty C., Pham T.D. (2022). Deep learning-based meta-classifier approach for COVID-19 classification using CT scan and chest X-ray images. Multimed. Syst..

[112] Chen H., Guo S., Hao Y., Fang Y., Fang Z., Wu W., Liu Z., Li S. (2021). Auxiliary Diagnosis for COVID-19 with Deep Transfer Learning. J. Digit. Imaging.

[113] Huang Z., Liu X., Wang R., Zhang M., Zeng X., Liu J., Yang Y., Liu X., Zheng H., Liang D. (2021). FaNet: Fast assessment network for the novel coronavirus (COVID-19) pneumonia based on 3D CT imaging and clinical symptoms. Appl. Intell..

[114] Jangam E., Barreto A.A.D., Annavarapu C.S.R. (2021). Automatic detection of COVID-19 from chest CT scan and chest X-rays images using deep learning, transfer learning and stacking. Appl. Intell..

[115] Singh V.K., Kolekar M.H. (2022). Deep learning empowered COVID-19 diagnosis using chest CT scan images for collaborative edge-cloud computing platform. Multimed. Tools Appl..

[116] Alirr O.I. (2021). Automatic deep learning system for COVID-19 infection quantification in chest CT. Multimed. Tools Appl..

[117] Kundu R., Singh P.K., Ferrara M., Ahmadian A., Sarkar R. (2021). ET-NET: An ensemble of transfer learning models for prediction of COVID-19 infection through chest CT-scan images. Multimed. Tools Appl..

[118] Saad W., Shalaby W.A., Shokair M., El-Samie F.A., Dessouky M., Abdellatef E. (2021). COVID-19 classification using deep feature concatenation technique. J. Ambient. Intell. Humaniz. Comput..

[119] Fung D.L., Liu Q., Zammit J., Leung C.K.S., Hu P. (2021). Self-supervised deep learning model for COVID-19 lung CT image segmentation highlighting putative causal relationship among age, underlying disease and COVID-19. J. Transl. Med..

[120] Tan W., Liu P., Li X., Liu Y., Zhou Q., Chen C., Gong Z., Yin X., Zhang Y. (2021). Classification of COVID-19 pneumonia from chest CT images based on reconstructed super-resolution images and VGG neural network. Health Inf. Sci. Syst..

[121] Lascu M.-R. (2021). Deep Learning in Classification of COVID-19 Coronavirus, Pneumonia and Healthy Lungs on CXR and CT Images. J. Med. Biol. Eng..

[122] Lassau N., Ammari S., Chouzenoux E., Gortais H., Herent P., Devilder M., Soliman S., Meyrignac O., Talabard M.-P., Lamarque J.-P. (2021). Integrating deep learning CT-scan model, biological and clinical variables to predict severity of COVID-19 patients. Nat. Commun..

[123] Pan F., Li L., Liu B., Ye T., Li L., Liu D., Ding Z., Chen G., Liang B., Yang L. (2021). A novel deep learning-based quantification of serial chest computed tomography in Coronavirus Disease 2019 (COVID-19). Sci. Rep..

[124] Yang D., Martinez C., Visuña L., Khandhar H., Bhatt C., Carretero J. (2021). Detection and analysis of COVID-19 in medical images using deep learning techniques. Sci. Rep..

[125] Gifani P., Shalbaf A., Vafaeezadeh M. (2021). Automated detection of COVID-19 using ensemble of transfer learning with deep convolutional neural network based on CT scans. Int. J. Comput. Assist. Radiol. Surg..

[126] Rahimzadeh M., Attar A., Sakhaei S.M. (2021). A fully automated deep learning-based network for detecting COVID-19 from a new and large lung CT scan dataset. Biomed. Signal Process. Control.

[127] Lee E.H., Zheng J., Colak E., Mohammadzadeh M., Houshmand G., Bevins N., Kitamura F., Altinmakas E., Reis E.P., Kim J.-K. (2021). Deep COVID DeteCT: An international experience on COVID-19 lung detection and prognosis using chest CT. NPJ Digit. Med..

[128] Mishra S. (2021). Deep Transfer Learning-Based Framework for COVID-19 Diagnosis Using Chest CT Scans and Clinical Information. SN Comput. Sci..

[129] Zhang Z., Ni X., Huo G., Li Q., Qi F. (2021). Novel coronavirus pneumonia detection and segmentation based on the deep-learning method. Ann. Transl. Med..

[130] Barbosa E.J.M., Gefter W.B., Ghesu F.C., Liu S., Mailhe B., Mansoor A., Grbic S., Vogt S. (2021). Automated Detection and Quantification of COVID-19 Airspace Disease on Chest Radiographs: A Novel Approach Achieving Expert Radiologist-Level Performance Using a Deep Convolutional Neural Network Trained on Digital Reconstructed Radiographs from Computed Tomography–Derived Ground Truth. Investig. Radiol..

[131] Zhao C., Xu Y., He Z., Tang J., Zhang Y., Han J., Shi Y., Zhou W. (2021). Lung segmentation and automatic detection of COVID-19 using radiomic features from chest CT images. Pattern Recognit..

[132] Jadhav S., Deng G., Zawin M., Kaufman A.E. (2021). COVID-view: Diagnosis of COVID-19 using Chest CT. IEEE Trans. Vis. Comput. Graph..

[133] Guiot J., Vaidyanathan A., Deprez L., Zerka F., Danthine D., Frix A.-N., Thys M., Henket M., Canivet G., Mathieu S. (2021). Development and Validation of an Automated Radiomic CT Signature for Detecting COVID-19. Diagnostics.

[134] Yao X.-J., Zhu Z.-Q., Wang S.-H., Zhang Y.-D. (2021). CSGBBNet: An Explainable Deep Learning Framework for COVID-19 Detection. Diagnostics.

[135] Singh G., Yow K.-C. (2021). Object or Background: An Interpretable Deep Learning Model for COVID-19 Detection from CT-Scan Images. Diagnostics.

[136] Zhu Z., Xingming Z., Tao G., Dan T., Li J., Chen X., Li Y., Zhou Z., Zhang X., Zhou J. (2021). Classification of COVID-19 by Compressed Chest CT Image through Deep Learning on a Large Patients Cohort. Interdiscip. Sci. Comput. Life Sci..

[137] Kuchana M., Srivastava A., Das R., Mathew J., Mishra A., Khatter K. (2021). AI aiding in diagnosing, tracking recovery of COVID-19 using deep learning on Chest CT scans. Multimed. Tools Appl..

[138] Khalifa N.E.M., Manogaran G., Taha M.H.N., Loey M. (2021). A deep learning semantic segmentation architecture for COVID-19 lesions discovery in limited chest CT datasets. Expert Syst..

[139] Bhuyan H.K., Chakraborty C., Shelke Y., Pani S.K. (2022). COVID-19 diagnosis system by deep learning approaches. Expert Syst..

[140] Heidarian S., Afshar P., Enshaei N., Naderkhani F., Rafiee M.J., Fard F.B., Samimi K., Atashzar S.F., Oikonomou A., Plataniotis K.N. (2021). COVID-FACT: A Fully-Automated Capsule Network-Based Framework for Identification of COVID-19 Cases from Chest CT Scans. Front. Artif. Intell..

[141] Ahsan M., Nazim R., Siddique Z., Huebner P. (2021). Detection of COVID-19 Patients from CT Scan and Chest X-ray Data Using Modified MobileNetV2 and LIME. Healthcare.

[142] Zhang Q., Chen Z., Liu G., Zhang W., Du Q., Tan J., Gao Q. (2021). Artificial Intelligence Clinicians Can Use Chest Computed Tomography Technology to Automatically Diagnose Coronavirus Disease 2019 (COVID-19) Pneumonia and Enhance Low-Quality Images. Infect. Drug Resist..

[143] Chaddad A., Hassan L., Desrosiers C. (2021). Deep CNN models for predicting COVID-19 in CT and X-ray images. J. Med. Imaging.

[144] Yousefzadeh M., Esfahanian P., Movahed S.M.S., Gorgin S., Rahmati D., Abedini A., Nadji S.A., Haseli S., Bakhshayesh Karam M., Kiani A. (2021). ai-corona: Radiologist-assistant deep learning framework for COVID-19 diagnosis in chest CT scans. PLoS ONE.

[145] Chen X., Yao L., Zhou T., Dong J., Zhang Y. (2021). Momentum contrastive learning for few-shot COVID-19 diagnosis from chest CT images. Pattern Recognit..

[146] Munusamy H., Muthukumar K.J., Gnanaprakasam S., Shanmugakani T.R., Sekar A. (2021). FractalCovNet architecture for COVID-19 Chest X-ray image Classification and CT-scan image Segmentation. Biocybern. Biomed. Eng..

[147] Wang S.-H., Nayak D.R., Guttery D.S., Zhang X., Zhang Y.-D. (2021). COVID-19 classification by CCSHNet with deep fusion using transfer learning and discriminant correlation analysis. Inf. Fusion.

[148] Jiang H., Tang S., Liu W., Zhang Y. (2021). Deep learning for COVID-19 chest CT (computed tomography) image analysis: A lesson from lung cancer. Comput. Struct. Biotechnol. J..

[149] Hu K., Huang Y., Huang W., Tan H., Chen Z., Zhong Z., Li X., Zhang Y., Gao X. (2021). Deep supervised learning using self-adaptive auxiliary loss for COVID-19 diagnosis from imbalanced CT images. Neurocomputing.

[150] Jingxin L., Mengchao Z., Yuchen L., Jinglei C., Yutong Z., Zhong Z., Lihui Z. (2022). COVID-19 lesion detection and segmentation–A deep learning method. Methods.

[151] Balaha H.M., El-Gendy E.M., Saafan M.M. (2021). CovH2SD: A COVID-19 detection approach based on Harris Hawks Optimization and stacked deep learning. Expert. Syst. Appl..

[152] Turkoglu M. (2021). COVID-19 Detection System Using Chest CT Images and Multiple Kernels-Extreme Learning Machine Based on Deep Neural Network. IRBM.

[153] Ahamed K.U., Islam M., Uddin A., Akhter A., Paul B.K., Abu Yousuf M., Uddin S., Quinn J.M., Moni M.A. (2021). A deep learning approach using effective preprocessing techniques to detect COVID-19 from chest CT-scan and X-ray images. Comput. Biol. Med..

[154] Pathan S., Siddalingaswamy P., Kumar P., Manohara Pai M.M., Ali T., Acharya U.R. (2021). Novel ensemble of optimized CNN and dynamic selection techniques for accurate COVID-19 screening using chest CT images. Comput. Biol. Med..

[155] Cruz J.F.H.S. (2021). An ensemble approach for multi-stage transfer learning models for COVID-19 detection from chest CT scans. Intell. Med..

[156] Hasan N.I. (2021). A hybrid method of COVID-19 patient detection from modified CT-scan/chest-X-ray images combining deep convolutional neural network and two-dimensional empirical mode decomposition. Comput. Methods Programs Biomed. Update.

[157] Abdel-Basset M., Hawash H., Moustafa N., Elkomy O.M. (2021). Two-Stage Deep Learning Framework for Discrimination between COVID-19 and Community-Acquired Pneumonia from Chest CT scans. Pattern Recognit. Lett..

[158] Fu Y., Xue P., Dong E. (2021). Densely connected attention network for diagnosing COVID-19 based on chest CT. Comput. Biol. Med..

[159] Aslan M.F., Unlersen M.F., Sabanci K., Durdu A. (2021). CNN-based transfer learning–BiLSTM network: A novel approach for COVID-19 infection detection. Appl. Soft Comput..

[160] Kundu R., Singh P.K., Mirjalili S., Sarkar R. (2021). COVID-19 detection from lung CT-Scans using a fuzzy integral-based CNN ensemble. Comput. Biol. Med..

[161] Müller D., Soto-Rey I., Kramer F. (2021). Robust chest CT image segmentation of COVID-19 lung infection based on limited data. Inform. Med. Unlocked.

[162] Li C., Yang Y., Liang H., Wu B. (2021). Transfer learning for establishment of recognition of COVID-19 on CT imaging using small-sized training datasets. Knowl.-Based Syst..

[163] Zhang Y.-D., Zhang Z., Zhang X., Wang S.-H. (2021). MIDCAN: A multiple input deep convolutional attention network for COVID-19 diagnosis based on chest CT and chest X-ray. Pattern Recognit. Lett..

[164] Xu X., Wen Y., Zhao L., Zhang Y., Zhao Y., Tang Z., Yang Z., Chen C.Y.C. (2021). CARes-UNet: Content-aware residual UNet for lesion segmentation of COVID-19 from chest CT images. Med. Phys..

[165] Mondal M.R.H., Bharati S., Podder P. (2021). CO-IRv2: Optimized InceptionResNetV2 for COVID-19 detection from chest CT images. PLoS ONE.

[166] Chen Y.M., Chen Y.J., Ho W.H., Tsai J.T. (2021). Classifying chest CT images as COVID-19 positive/negative using a convolutional neural network ensemble model and uniform experimental design method. BMC Bioinform..

[167] Alshazly H., Linse C., Barth E., Martinetz T. (2021). Explainable COVID-19 Detection Using Chest CT Scans and Deep Learning. Sensors.

[168] Voulodimos A., Protopapadakis E., Katsamenis I., Doulamis A., Doulamis N. (2021). A Few-Shot U-Net Deep Learning Model for COVID-19 Infected Area Segmentation in CT Images. Sensors.

[169] Khan M.A., Alhaisoni M., Tariq U., Hussain N., Majid A., Damaševičius R., Maskeliūnas R. (2021). COVID-19 Case Recognition from Chest CT Images by Deep Learning, Entropy-Controlled Firefly Optimization, and Parallel Feature Fusion. Sensors.

[170] Rajasekar S.J.S., Narayanan V., Perumal V. (2021). Detection of COVID-19 from Chest CT Images Using CNN with MLP Hybrid Model. pHealth.

[171] Xie Q., Lu Y., Xie X., Mei N., Xiong Y., Li X., Zhu Y., Xiao A., Yin B. (2021). The usage of deep neural network improves distinguishing COVID-19 from other suspected viral pneumonia by clinicians on chest CT: A real-world study. Eur. Radiol..

[172] Sethy P.K., Behera S.K., Anitha K., Pandey C., Khan M. (2021). Computer aid screening of COVID-19 using X-ray and CT scan images: An inner comparison. J. X-ray Sci. Technol..

[173] Özyurt F. (2021). Automatic Detection of COVID-19 Disease by Using Transfer Learning of Light Weight Deep Learning Model. Trait. Du Signal.

[174] Garain A., Basu A., Giampaolo F., Velasquez J.D., Sarkar R. (2021). Detection of COVID-19 from CT scan images: A spiking neural network-based approach. Neural Comput. Appl..

[175] Elghamrawy S.M., Hassnien A.E., Snasel V. (2021). Optimized Deep Learning-Inspired Model for the Diagnosis and Prediction of COVID-19. Comput. Mater. Contin..

[176] Sen S., Saha S., Chatterjee S., Mirjalili S., Sarkar R. (2021). A bi-stage feature selection approach for COVID-19 prediction using chest CT images. Appl. Intell..

[177] Teodoro A.A.M., Silva D.H., Saadi M., Okey O.D., Rosa R.L., Al Otaibi S., Rodríguez D.Z. (2021). An Analysis of Image Features Extracted by CNNs to Design Classification Models for COVID-19 and Non-COVID-19. J. Signal Process. Syst..

[178] Yasar H., Ceylan M. (2021). Deep Learning–Based Approaches to Improve Classification Parameters for Diagnosing COVID-19 from CT Images. Cogn. Comput..

[179] Ibrahim M.R., Youssef S.M., Fathalla K.M. (2021). Abnormality detection and intelligent severity assessment of human chest computed tomography scans using deep learning: A case study on SARS-COV-2 assessment. J. Ambient. Intell. Humaniz. Comput..

[180] Afshar P., Heidarian S., Enshaei N., Naderkhani F., Rafiee M.J., Oikonomou A., Fard F.B., Samimi K., Plataniotis K.N., Mohammadi A. (2021). COVID-CT-MD, COVID-19 computed tomography scan dataset applicable in machine learning and deep learning. Sci. Data.

[181] Liu B., Liu P., Dai L., Yang Y., Xie P., Tan Y., Du J., Shan W., Zhao C., Zhong Q. (2021). Assisting scalable diagnosis automatically via CT images in the combat against COVID-19. Sci. Rep..

[182] Kundu R., Basak H., Singh P.K., Ahmadian A., Ferrara M., Sarkar R. (2021). Fuzzy rank-based fusion of CNN models using Gompertz function for screening COVID-19 CT-scans. Sci. Rep..

[183] Pal B., Gupta D., Mahfuz R.A., Alyami S.A., Moni M.A. (2021). Vulnerability in Deep Transfer Learning Models to Adversarial Fast Gradient Sign Attack for COVID-19 Prediction from Chest Radiography Images. Appl. Sci..

[184] Biswas S., Chatterjee S., Majee A., Sen S., Schwenker F., Sarkar R. (2021). Prediction of COVID-19 from Chest CT Images Using an Ensemble of Deep Learning Models. Appl. Sci..

[185] Helwan A., Ma’aitah M.K.S., Hamdan H., Ozsahin D.U., Tuncyurek O. (2021). Radiologists versus Deep Convolutional Neural Networks: A Comparative Study for Diagnosing COVID-19. Comput. Math. Methods Med..

[186] Castiglione A., Vijayakumar P., Nappi M., Sadiq S., Umer M. (2021). COVID-19: Automatic Detection of the Novel Coronavirus Disease From CT Images Using an Optimized Convolutional Neural Network. IEEE Trans. Ind. Inform..

[187] Yan Q., Wang B., Gong D., Luo C., Zhao W., Shen J., Ai J., Shi Q., Zhang Y., Jin S. (2021). COVID-19 Chest CT Image Segmentation Network by Multi-Scale Fusion and Enhancement Operations. IEEE Trans. Big Data.

[188] Suri J.S., Agarwal S., Pathak R., Ketireddy V., Columbu M., Saba L., Gupta S.K., Faa G., Singh I.M., Turk M. (2021). COVLIAS 1.0: Lung Segmentation in COVID-19 Computed Tomography Scans Using Hybrid Deep Learning Artificial Intelligence Models. Diagnostics.

[189] Nair R., Alhudhaif A., Koundal D., Doewes R.I., Sharma P. (2021). Deep learning-based COVID-19 detection system using pulmonary CT scans. Turk. J. Electr. Eng. Comput. Sci..

[190] Wan Y., Zhou H., Zhang X. (2021). An Interpretation Architecture for Deep Learning Models with the Application of COVID-19 Diagnosis. Entropy.

[191] Guo X., Lei Y., He P., Zeng W., Yang R., Ma Y., Feng P., Lyu Q., Wang G., Shan H. (2021). An ensemble learning method based on ordinal regression for COVID-19 diagnosis from chest CT. Phys. Med. Biol..

[192] Xia Y., Chen W., Ren H., Zhao J., Wang L., Jin R., Zhou J., Wang Q., Yan F., Zhang B. (2021). A rapid screening classifier for diagnosing COVID-19. Int. J. Biol. Sci..

[193] Polat H., Özerdem M.S., Ekici F., Akpolat V. (2021). Automatic detection and localization of COVID-19 pneumonia using axial computed tomography images and deep convolutional neural networks. Int. J. Imaging Syst. Technol..

[194] Li X., Tan W., Liu P., Zhou Q., Yang J. (2021). Classification of COVID-19 Chest CT Images Based on Ensemble Deep Learning. J. Healthc. Eng..

[195] Owais M., Baek N.R., Park K.R. (2021). Domain-Adaptive Artificial Intelligence-Based Model for Personalized Diagnosis of Trivial Lesions Related to COVID-19 in Chest Computed Tomography Scans. J. Pers. Med..

[196] Jia G., Lam H.-K., Xu Y. (2021). Classification of COVID-19 chest X-ray and CT images using a type of dynamic CNN modification method. Comput. Biol. Med..

[197] He K., Zhao W., Xie X., Ji W., Liu M., Tang Z., Shi Y., Shi F., Gao Y., Liu J. (2021). Synergistic learning of lung lobe segmentation and hierarchical multi-instance classification for automated severity assessment of COVID-19 in CT images. Pattern Recognit..

[198] Murugan R., Goel T., Mirjalili S., Chakrabartty D.K. (2021). WOANet: Whale optimized deep neural network for the classification of COVID-19 from radiography images. Biocybern. Biomed. Eng..

[199] Naeem H., Bin-Salem A.A. (2021). A CNN-LSTM network with multi-level feature extraction-based approach for automated detection of coronavirus from CT scan and X-ray images. Appl. Soft Comput..

[200] Kalane P., Patil S., Patil B., Sharma D.P. (2021). Automatic detection of COVID-19 disease using U-Net architecture based fully convolutional network. Biomed. Signal Process. Control..

[201] Fouladi S., Ebadi M., Safaei A.A., Bajuri M.Y., Ahmadian A. (2021). Efficient deep neural networks for classification of COVID-19 based on CT images: Virtualization via software defined radio. Comput. Commun..

[202] Wang S.-H., Govindaraj V.V., Górriz J.M., Zhang X., Zhang Y.-D. (2021). COVID-19 classification by FGCNet with deep feature fusion from graph convolutional network and convolutional neural network. Inf. Fusion.

[203] Yu X., Lu S., Guo L., Wang S.H., Zhang Y.D. (2021). ResGNet-C: A graph convolutional neural network for detection of COVID-19. Neurocomputing.

[204] Gao K., Su J., Jiang Z., Zeng L.-L., Feng Z., Shen H., Rong P., Xu X., Qin J., Yang Y. (2021). Dual-branch combination network (DCN): Towards accurate diagnosis and lesion segmentation of COVID-19 using CT images. Med. Image Anal..

[205] Sahoo P., Roy I., Ahlawat R., Irtiza S., Khan L. (2021). Potential diagnosis of COVID-19 from chest X-ray and CT findings using semi-supervised learning. Phys. Eng. Sci. Med..

[206] Lacerda P., Barros B., Albuquerque C., Conci A. (2021). Hyperparameter Optimization for COVID-19 Pneumonia Diagnosis Based on Chest CT. Sensors.

[207] Siddiqui S.Y., Abbas S., Khan M.A., Naseer I., Masood T., Khan K.M., Al Ghamdi M.A., Almotiri S.H. (2021). Intelligent Decision Support System for COVID-19 Empowered with Deep Learning. Comput. Mater. Contin..

[208] Haikel A. (2021). CNN ensemble approach to detect COVID-19 from computed tomography chest images. Comput. Mater. Contin..

[209] Bekhet S., Alkinani M.H., Tabares-Soto R., Hassaballah M. (2021). An Efficient Method for COVID-19 Detection Using Light Weight Convolutional Neural Network. Comput. Mater. Contin..

[210] Kaushik H., Singh D., Tiwari S., Kaur M., Jeong C.-W., Nam Y., Khan M.A. (2021). Screening of COVID-19 Patients Using Deep Learning and IoT Framework. Comput. Mater. Contin..

[211] El-Shafai W., Ali A.M., El-Rabaie E.-S.M., Soliman N.F., Algarni A.D., El-Samie F.E.A. (2021). Automated COVID-19 Detection Based on Single-Image Super-Resolution and CNN Models. Comput. Mater. Contin..

[212] Masud M., Alshehri M.D., Alroobaea R., Shorfuzzaman M. (2021). Leveraging Convolutional Neural Network for COVID-19 Disease Detection Using CT Scan Images. Intell. Autom. Soft Comput..

[213] El-Shafai W., El-Hag N.A., El-Banby G.M., Khalaf A.A.M., Soliman N.F., Algarni A.D., El-Samie F.E.A. (2021). An Efficient CNN-Based Automated Diagnosis Framework from COVID-19 CT Images. Comput. Mater. Contin..

[214] Kassania S.H., Kassanib P.H., Wesolowskic M.J., Schneidera K.A., Detersa R. (2021). Automatic Detection of Coronavirus Disease (COVID-19) in X-ray and CT Images: A Machine Learning Based Approach. Biocybern. Biomed. Eng..

[215] Wang B., Jin S., Yan Q., Xu H., Luo C., Wei L., Zhao W., Hou X., Ma W., Xu Z. (2021). AI-assisted CT imaging analysis for COVID-19 screening: Building and deploying a medical AI system. Appl. Soft Comput..

[216] Ahuja S., Panigrahi B.K., Dey N., Rajinikanth V., Gandhi T.K. (2021). Deep transfer learning-based automated detection of COVID-19 from lung CT scan slices. Appl. Intell..

[217] Pu J., Leader J.K., Bandos A., Ke S., Wang J., Shi J., Du P., Guo Y., Wenzel S.E., Fuhrman C.R. (2021). Automated quantification of COVID-19 severity and progression using chest CT images. Eur. Radiol..

[218] Maghdid H.S., Asaad A.T., Ghafoor K.Z.G., Sadiq A.S., Mirjalili S., Khan M.K.K. (2021). Diagnosing COVID-19 pneumonia from X-ray and CT images using deep learning and transfer learning algorithms. Multimodal Image Exploitation and Learning 2021.

[219] Kumar I., Alshamrani S.S., Kumar A., Rawat J., Singh K.U., Rashid M., AlGhamdi A.S. (2021). Deep Learning Approach for Analysis and Characterization of COVID-19. Comput. Mater. Contin..

[220] Wang S., Kang B., Ma J., Zeng X., Xiao M., Guo J., Cai M., Yang J., Li Y., Meng X. (2021). A deep learning algorithm using CT images to screen for Corona Virus Disease (COVID-19). Eur. Radiol..

[221] Khurana Y., Soni U. (2022). Leveraging deep learning for COVID-19 diagnosis through chest imaging. Neural Comput. Appl..

[222] Canayaz M., Şehribanoğlu S., Özdağ R., Demir M. (2022). COVID-19 diagnosis on CT images with Bayes optimization-based deep neural networks and machine learning algorithms. Neural Comput. Appl..

[223] Subhalakshmi R.T., Balamurugan S.A.A., Sasikala S. (2022). Deep learning based fusion model for COVID-19 diagnosis and classification using computed tomography images. Concurr. Eng..

[224] Zouch W., Sagga D., Echtioui A., Khemakhem R., Ghorbel M., Mhiri C., Ben Hamida A. (2022). Detection of COVID-19 from CT and Chest X-ray Images Using Deep Learning Models. Ann. Biomed. Eng..

[225] Balaha H.M., El-Gendy E.M., Saafan M.M. (2022). A complete framework for accurate recognition and prognosis of COVID-19 patients based on deep transfer learning and feature classification approach. Artif. Intell. Rev..

[226] Habib M., Ramzan M., Khan S.A. (2022). A Deep Learning and Handcrafted Based Computationally Intelligent Technique for Effective COVID-19 Detection from X-ray/CT-scan Imaging. J. Grid Comput..

[227] Montalbo F.J. (2022). Truncating fined-tuned vision-based models to lightweight deployable diagnostic tools for SARS-CoV-2 infected chest X-rays and CT-scans. Multimed. Tools Appl..

[228] Ali A.M., Ghafoor K., Mulahuwaish A., Maghdid H. (2022). COVID-19 pneumonia level detection using deep learning algorithm and transfer learning. Evol. Intell..

[229] Pandey S.K., Bhandari A.K., Singh H. (2022). A transfer learning based deep learning model to diagnose COVID-19 CT scan images. Health Technol..

[230] Liu B., Nie X., Li Z., Yang S., Tian Y. (2022). Evolving deep convolutional neural networks by IP-based marine predator algorithm for COVID-19 diagnosis using chest CT scans. J. Ambient. Intell. Humaniz. Comput..

[231] Luo J., Sun Y., Chi J., Liao X., Xu C. (2022). A novel deep learning-based method for COVID-19 pneumonia detection from CT images. BMC Med. Inform. Decis. Mak..

[232] Saheb S.K., Narayanan B., Rao T.V.N. (2022). ADL-CDF: A Deep Learning Framework for COVID-19 Detection from CT Scans Towards an Automated Clinical Decision Support System. Arab. J. Sci. Eng..

[233] Khurana Batra P., Aggarwal P., Wadhwa D., Gulati M. (2022). Predicting pattern of coronavirus using X-ray and CT scan images. Netw. Model. Anal. Health Inform. Bioinform..

[234] Cao Y., Zhang C., Peng C., Zhang G., Sun Y., Jiang X., Wang Z., Zhang D., Wang L., Liu J. (2022). A convolutional neural network-based COVID-19 detection method using chest CT images. Ann. Transl. Med..

[235] Yazdani A., Fekri-Ershad S., Jelvay S. (2022). Diagnosis of COVID-19 Disease in Chest CT-Scan Images Based on Combination of Low-Level Texture Analysis and MobileNetV2 Features. Comput. Intell. Neurosci..

[236] Ibrahim D.A., Zebari D.A., Mohammed H.J., Mohammed M.A. (2022). Effective hybrid deep learning model for COVID-19 patterns identification using CT images. Expert Syst..

[237] Akinyelu A.A., Blignaut P. (2022). COVID-19 diagnosis using deep learning neural networks applied to CT images. Front. Artif. Intell..

[238] Florescu L.M., Streba C.T., Şerbănescu M.-S., Mămuleanu M., Florescu D.N., Teică R.V., Nica R.E., Gheonea I.A. (2022). Federated Learning Approach with Pre-Trained Deep Learning Models for COVID-19 Detection from Unsegmented CT images. Life.

[239] Baghdadi N.A., Malki A., Abdelaliem S.F., Balaha H.M., Badawy M., Elhosseini M. (2022). An automated diagnosis and classification of COVID-19 from chest CT images using a transfer learning-based convolutional neural network. Comput. Biol. Med..

[240] Shaik N.S., Cherukuri T.K. (2022). Transfer learning based novel ensemble classifier for COVID-19 detection from chest CT-scans. Comput. Biol. Med..

[241] Reis H.C., Turk V. (2022). COVID-DSNet: A novel deep convolutional neural network for detection of coronavirus (SARS-CoV-2) cases from CT and Chest X-ray images. Artif. Intell. Med..

[242] Garg A., Salehi S., La Rocca M., Garner R., Duncan D. (2022). Efficient and visualizable convolutional neural networks for COVID-19 classification using Chest CT. Expert Syst. Appl..

[243] Fan X., Feng X., Dong Y., Hou H. (2022). COVID-19 CT image recognition algorithm based on transformer and CNN. Displays.

[244] Karthik R., Menaka R., Hariharan M., Won D. (2022). CT-based severity assessment for COVID-19 using weakly supervised non-local CNN. Appl. Soft Comput..

[245] Verma A., Amin S.B., Naeem M., Saha M. (2022). Detecting COVID-19 from chest computed tomography scans using AI-driven android application. Comput. Biol. Med..

[246] Abugabah A., Al-Smadi A.M., Almotairi S. (2022). SEL-COVIDNET: An intelligent application for the diagnosis of COVID-19 from chest X-rays and CT-scans. Inform. Med. Unlocked.

[247] Fallahpoor M., Chakraborty S., Heshejin M.T., Chegeni H., Horry M.J., Pradhan B. (2022). Generalizability assessment of COVID-19 3D CT data for deep learning-based disease detection. Comput. Biol. Med..

[248] Sadik F., Dastider A.G., Subah M.R., Mahmud T., Fattah S.A. (2022). A dual-stage deep convolutional neural network for automatic diagnosis of COVID-19 and pneumonia from chest CT images. Comput. Biol. Med..

[249] Huang M.-L., Liao Y.-C. (2022). A lightweight CNN-based network on COVID-19 detection using X-ray and CT images. Comput. Biol. Med..

[250] Li C.F., Xu Y.D., Ding X.H., Zhao J.J., Du R.Q., Wu L.Z., Sun W.P. (2022). MultiR-Net: A Novel Joint Learning Network for COVID-19 segmentation and classification. Comput. Biol. Med..

[251] Hemalatha M. (2022). A hybrid random forest deep learning classifier empowered edge cloud architecture for COVID-19 and pneumonia detection. Expert Syst. Appl..

[252] Wang X., Yuan Y., Guo D., Huang X., Cui Y., Xia M., Wang Z., Bai C., Chen S. (2022). SSA-Net: Spatial self-attention network for COVID-19 pneumonia infection segmentation with semi-supervised few-shot learning. Med. Image Anal..

[253] Qi Q., Qi S., Wu Y., Li C., Tian B., Xia S., Ren J., Yang L., Wang H., Yu H. (2022). Fully automatic pipeline of convolutional neural networks and capsule networks to distinguish COVID-19 from community-acquired pneumonia via CT images. Comput. Biol. Med..

[254] Oğuz Ç., Yağanoğlu M. (2022). Detection of COVID-19 using deep learning techniques and classification methods. Inf. Process. Manag..

[255] Yang L., Wang S.-H., Zhang Y.-D. (2022). EDNC: Ensemble Deep Neural Network for COVID-19 Recognition. Tomography.

[256] Tello-Mijares S., Woo F. (2022). Novel COVID-19 Diagnosis Delivery App Using Computed Tomography Images Analyzed with Saliency-Preprocessing and Deep Learning. Tomography.

[257] Heidari A., Toumaj S., Navimipour N.J., Unal M. (2022). A privacy-aware method for COVID-19 detection in chest CT images using lightweight deep conventional neural network and blockchain. Comput. Biol. Med..

[258] Ortiz A., Trivedi A., Desbiens J., Blazes M., Robinson C., Gupta S., Dodhia R., Bhatraju P.K., Liles W.C., Lee A. (2022). Effective deep learning approaches for predicting COVID-19 outcomes from chest computed tomography volumes. Sci. Rep..

[259] Sangeetha S.K.B., Kumar M.S., Deeba K., Rajadurai H., Maheshwari V., Dalu G.T. (2022). An Empirical Analysis of an Optimized Pretrained Deep Learning Model for COVID-19 Diagnosis. Comput. Math. Methods Med..

[260] Mohammed M.A., Al-Khateeb B., Yousif M., Mostafa S.A., Kadry S., Abdulkareem K.H., Garcia-Zapirain B. (2022). Novel Crow Swarm Optimization Algorithm and Selection Approach for Optimal Deep Learning COVID-19 Diagnostic Model. Comput. Intell. Neurosci..

[261] Joshi A.M., Nayak D.R. (2022). MFL-Net: An Efficient Lightweight Multi-Scale Feature Learning CNN for COVID-19 Diagnosis From CT Images. IEEE J. Biomed. Health Inform..

[262] Zhang S., Yuan G.-C. (2022). Deep Transfer Learning for COVID-19 Detection and Lesion Recognition Using Chest CT Images. Comput. Math. Methods Med..

[263] Mouhafid M., Salah M., Yue C., Xia K. (2022). Deep Ensemble Learning-Based Models for Diagnosis of COVID-19 from Chest CT Images. Healthcare.

[264] Dara S., Kanapala A., Babu A.R., Dhamercherala S., Vidyarthi A., Agarwal R. (2022). Scalable Federated-Learning and Internet-of-Things enabled architecture for Chest Computer Tomography image classification. Comput. Electr. Eng..

[265] Özdemir Ö., Sönmez E.B. (2022). Attention mechanism and mixup data augmentation for classification of COVID-19 Computed Tomography images. J. King Saud Univ.—Comput. Inf. Sci..

[266] Ahuja S., Panigrahi B.K., Dey N., Taneja A., Gandhi T.K. (2022). McS-Net: Multi-class Siamese network for severity of COVID-19 infection classification from lung CT scan slices. Appl. Soft Comput..

[267] Messaoud S., Bouaafia S., Maraoui A., Khriji L., Ammari A.C., Machhout M. (2022). Detection of COVID-19 and other pneumonia cases from CT and X-ray chest images using deep learning based on feature reuse residual block and depthwise dilated convolutions neural network. Can. J. Infect. Dis. Med. Microbiol..

[268] Manconi A., Armano G., Gnocchi M., Milanesi L. (2022). A Soft-Voting Ensemble Classifier for Detecting Patients Affected by COVID-19. Appl. Sci..

[269] Chen C., Li R., Shen H., Xia L. (2022). Long Short-Term Memory Based Framework for Longitudinal Assessment of COVID-19 Using CT Imaging and Laboratory Data. IEEE Access.

[270] Lu S.-Y., Zhang Z., Zhang Y.-D., Wang S.-H. (2022). CGENet: A Deep Graph Model for COVID-19 Detection Based on Chest CT. Biology.

[271] Owais M., Sultan H., Baek N.R., Lee Y.W., Usman M., Nguyen D.T., Batchuluun G., Park K.R. (2022). Deep 3D Volumetric Model Genesis for Efficient Screening of Lung Infection Using Chest CT Scans. Mathematics.

[272] Yoo S.-J., Qi X., Inui S., Kim H., Jeong Y.J., Lee K.H., Lee Y.K., Lee B.Y., Kim J.Y., Jin K.N. (2022). Deep Learning–Based Automatic CT Quantification of Coronavirus Disease 2019 Pneumonia: An International Collaborative Study. J. Comput. Assist. Tomogr..

[273] Suri J.S., Agarwal S., Chabert G.L., Carriero A., Paschè A., Danna P.S.C., Saba L., Mehmedović A., Faa G., Singh I.M. (2022). COVLIAS 1.0 Lesion vs. MedSeg: An Artificial Intelligence Framework for Automated Lesion Segmentation in COVID-19 Lung Computed Tomography Scans. Diagnostics.

[274] Ghose P., Alavi M., Tabassum M., Uddin A., Biswas M., Mahbub K., Gaur L., Mallik S., Zhao Z. (2022). Detecting COVID-19 infection status from chest X-ray and CT scan via single transfer learning-driven approach. Front. Genet..

[275] Gunraj H., Sabri A., Koff D., Wong A. (2022). COVID-Net CT-2: Enhanced Deep Neural Networks for Detection of COVID-19 From Chest CT Images Through Bigger, More Diverse Learning. Front. Med..

[276] Yousefzadeh M., Hasanpour M., Zolghadri M., Salimi F., Vaziri A.Y., Abadi A.M.A., Jafari R., Esfahanian P., Nazem-Zadeh M.-R. (2022). Deep learning framework for prediction of infection severity of COVID-19. Front. Med..

[277] Choudhary T., Gujar S., Goswami A., Mishra V., Badal T. (2022). Deep learning-based important weights-only transfer learning approach for COVID-19 CT-scan classification. Appl. Intell..

[278] Chouat I., Echtioui A., Khemakhem R., Zouch W., Ghorbel M., Ben Hamida A. (2022). COVID-19 detection in CT and CXR images using deep learning models. Biogerontology.

[279] Dialameh M., Hamzeh A., Rahmani H., Radmard A.R., Dialameh S. (2022). Proposing a novel deep network for detecting COVID-19 based on chest images. Sci. Rep..

[280] Venkatachalam K., Siuly S., Kumar M.V., Lalwani P., Mishra M.K., Kabir E. (2022). A Hybrid Approach for COVID-19 Detection Using Biogeography-Based Optimization and Deep Learning. Comput. Mater. Contin..

[281] Latif G., Morsy H., Hassan A., Alghazo J. (2022). Novel Coronavirus and Common Pneumonia Detection from CT Scans Using Deep Learning-Based Extracted Features. Viruses.

[282] El-Shafai W., Mahmoud A.A., El-Rabaie E.-S.M., Taha T.E., Zahran O.F., El-Fishawy A.S., Abd-Elnaby M., El-Samie F.E.A. (2022). Efficient Deep CNN Model for COVID-19 Classification. Comput. Mater. Contin..

[283] Xue Y., Onzo B.-M., Mansour R.F., Su S. (2022). Deep Convolutional Neural Network Approach for COVID-19 Detection. Comput. Syst. Sci. Eng..

[284] El-Shafai W., Algarni A.D., El Banby G.M., El-Samie F.E.A., Soliman N.F. (2022). Classification Framework for COVID-19 Diagnosis Based on Deep CNN Models. Intell. Autom. Soft Comput..

[285] Santosh K., Ghosh S. (2021). COVID-19 Imaging Tools: How Big Data is Big?. J. Med. Syst..

[286] Santosh K.C. (2020). AI-Driven Tools for Coronavirus Outbreak: Need of Active Learning and Cross-Population Train/Test Models on Multitudinal/Multimodal Data. J. Med. Syst..

